# Filling the gaps in Australian angiosperm genomes

**DOI:** 10.1371/journal.pone.0359340

**Published:** 2026-11-04

**Authors:** Theodore R. Allnutt, Todd G. B. McLay, Ashley Jones, Richard Edwards, Gareth D. Holmes, Alexander N. Schmidt-Lebuhn, Rose L. Andrew, Rachael M. Fowler, Chris Jackson, Mabel Lum, Jeremy J. Bruhl, Scott Ferguson, Alexander Fournier-Level, Matthew G. Lewsey, Michael Roach, Tareg Shaldoom, David J. Cantrill

**Affiliations:** 1 Royal Botanic Gardens Victoria, Birdwood Avenue, Melbourne, Victoria, Australia; 2 National Biodiversity DNA Library, National Research Collections Australia, CSIRO, Parkville, Victoria, Australia; 3 Research School of Biology, The Australian National University, Canberra, Australian Capital Territory, Australia; 4 Oceans institute, The University of Western Australia, Perth, Western Australia, Australia; 5 Evolution & Ecology Research Centre, School of Biotechnology and Biomolecular Sciences, University of New South Wales, Sydney, New South Wales, Australia; 6 CSIRO, Centre for Australian National Biodiversity Research (a joint venture of CSIRO and Parks Australia), Canberra, Australian Capital Territory, Australia; 7 N.C.W. Beadle Herbarium and School of Environmental and Rural Science, University of New England, Armidale, New South Wales, Australia; 8 School of BioSciences, The University of Melbourne, Parkville, Victoria, Australia; 9 Bioplatforms Australia Ltd., Sydney, New South Wales, Australia; 10 Department of Integrative Biology, University of California, Berkeley, California, United States of America; 11 La Trobe Institute for Sustainable Agriculture and Food, La Trobe University, Bundoora, Victoria, Australia; 12 Flinders Health and Medical Research Institute, Flinders University, Adelaide, South Australia, Australia; South China Agricultural University, CHINA

## Abstract

The Genomics for Australian Plants Initiative (GAP) reference genome project aimed to provide high quality angiosperm genomes for previously underrepresented taxonomic groups with evolutionary significance, cultural value, biogeographic importance, and concerning conservation status in Australia. Here we present the results of long-read genomic sequencing and assembly for 29 selected angiosperm species, including analyses of genome completeness, contiguity, ploidy, gene annotations, and repeat content. Plastid and mitochondrion genomes were also assembled and annotated. The data provided a diverse selection of genome size, ploidy and complexity, which were compared. The long read sequencing methods, PacBio HiFi, and Oxford Nanopore Technologies (ONT), used in tandem and individually where appropriate, provided high quality near complete telomere to telomere assemblies. These genomes provide valuable genomic resources for Australian plants that will enable and extend research in evolution, conservation, functional adaptation, and comparative genomics.

## Introduction

Over the past twenty years, whole-genome sequencing has transformed plant science, especially in horticultural applications such as crop improvement and breeding [1]. Early efforts focused predominantly on model and crop species [2], leading to uneven representation across flowering plant diversity. Although large-scale initiatives such as the 1,000 Plant Genomes Project (1KP, 2019) and the Earth BioGenome Project (EBP; [3]) have improved sequencing across broad phylogenetic scales, most genomic resources are still concentrated in economically important groups [4]. The development of third-generation, long-read sequencing has made obtaining high-quality genomes much more practical and financially achievable for non-model/non-commercial plants, enabling the investigation of genomes for understanding evolution, including genome structure, adaptation, and functional diversity.

The Australian flora represents highly distinctive continental radiations, characterised by high levels of endemism and extensive diversification across arid, nutrient-poor, and fire-prone environments [5]. Despite this evolutionary and ecological significance, genomic resources for native Australian angiosperms have historically been sparse and phylogenetically uneven, often concentrated in crop relatives (e.g., *Oryza*; [6]; *Citrus* [7],) or commercially important plantation taxa (e.g., *Eucalyptus*; [8–10]). Much of Australia’s native plant diversity still lacks genomic data, despite the role genome-scale data now play in conservation, ecology, and evolutionary biology.

The Genomics for Australian Plants Initiative (GAP) was established to address this disparity by coordinating a partnership between national plant collections and sharing funding across Australian herbaria and botanic gardens, supported by shared technical expertise in DNA extraction and genome assembly [11]. A detailed rationale for the choice of each species and background information is given in Table 1. GAP focused on lineages underrepresented in global databases, including phylogenetically distinct groups and species of conservation or evolutionary significance, e.g., [12,13]. By using curated collections and a coordinated national framework. The first phase of GAP produced three genomes: *Acacia pycnantha*, golden wattle, the Australian national floral emblem; *Telopea speciosissima*, waratah, the floral emblem of New South Wales, and *Tasmannia lanceolata* [12,13], (https://www.ncbi.nlm.nih.gov/datasets/genome/GCF_043159265.1/). Knowledge gained in the first phase in sampling, DNA purification, sequence library preparation, long-read sequencing, and bioinformatics was then exploited in the consortium for the further 29 genomes presented here which span 24 orders and 29 families, including 3 orders lacking any previously published genomic resources (Austrobaileyales, Berberidopsidales, and Paracryphiales). These reference genomes are high-quality long-read genome assemblies with *in silico* annotations. Assembly size and contiguity were evaluated against genome size estimates from flow cytometry, concordance between expected and observed ploidy was assessed, and genome completeness, telomere recovery, repeat composition, and gene content were characterised. Plastid and mitochondrial genomes were also assembled and annotated where possible. All sequence data, bioinformatic workflows, and specimen metadata are publicly available.

**Table 1:** Background information and rationale for species selection.

Species	Rationale
*Archirhodomyrtus beckleri*	*Archirhodomyrtus beckleri* is one of a number of rainforest Myrtaceae that are at risk of extinction from the incursion and establishment of myrtle rust in Australia. Considerable research is being done to understand these susceptible taxa and identify potential mechanisms of resistance to this pathogen [14].
*Austrobaileya scandens*	*Austrobaileya scandens* is part of the ANITA grade at the base of the angiosperm tree and occupies a key position in understanding the evolution of the group [15]. Currently there are no reference genomes for this order.0
*Bellendena montana*	This monotypic genus is identified as the sister taxa to the rest of the Proteaceae or sometimes sister to the Persoonioideae subfamily [16]. Study of the genus would improve our understanding of the continental radiation of these taxa.
*Callicoma serratifolia*	This species occupies mesic forest habitats and represents an important lineage for understanding the evolutionary radiation of woody Australian plants [17]. The Cunoniaceae have diverse floral morphologies, and their study will help resolve phylogenetic relationships within this largely understudied family.
*Dampiera purpurea*	A member of Goodeniaceae, an almost exclusively Australian family with high morphological diversity [18]. The genus *Dampiera* contains over 70 species and is an important component of Australian shrublands and heathlands. Goodeniaceae flowers exhibit unique pollen presentation mechanisms [19]. No reference genome is currently available in the genus..
*Dendrocnide excelsa*	This species is a representative of the nettle family (Urticaceae) and the genome provides a reference for a previously unsampled genus. *Dendrocnide excelsa* (Giant Stinging Tree) and its sister species *D. moroides* (Gympie-Gympie) have considerable potential for therapeutic agents related to their stinging toxins [20].
*Denhamia bilocularis*	A member of Celastraceae, a diverse and large family in Australian floras, this species is found in eastern Australian forests and woodlands, contributing to the diverse understory vegetation of these ecosystems. The Celastraceae include many species with bioactive compounds. No reference genome is available in the genus.
*Doryanthes excelsa*	The Gymea Lily is a charismatic species from the Doryanthaceae, a small family endemic to eastern Australia. This iconic plant produces one of the largest inflorescences of any Australian plant and has cultural significance to Indigenous peoples, who traditionally harvested the flower stems and roots as food [21]. The family has an ambiguous phylogenetic position within the angiosperms, and genomic data will help resolve its placement [22]. Its distinctive growth form with massive rosettes makes it an important species for studying convergent evolution with other large monocots. There is no reference genome available in this family.
*Elaeocarpus reticulatus*	Blueberry Ash represents the first genome sequence for Elaeocarpaceae, a family with a Gondwanan distribution and important presence in Australian rainforests. This species is valued horticulturally for its attractive blue fruits and fringed white flowers, and it is a common component of eastern Australian coastal vegetation. Elaeocarpaceae have diverse fruit types and seed dispersal mechanisms. The family also includes many rainforest species of conservation concern [23].
*Eremophila drummondii*	*Eremophila* (Emu bush) are a large and diverse Australian endemic genus of >250 species. The genus has played a significant role in traditional Aboriginal medicine, and it is at the center of current chemical research for the purposes of natural product discovery and pharmaceutical development. *Eremophila drummondii* is native to the south-west of Western Australia and is placed in a morphologically diverse lineage that contains a number of species in need of conservation management, and species containing known bioactive constituents. This genome marks the first genomic resource within *Eremophila* and the Australasian tribe Myoporeae, family Scrophulariaceae.
*Eucryphia lucida*	Leatherwood is endemic to Tasmania and produces highly valued monofloral honey that supports significant apiary industries. As the second Cunoniaceae genome in this study, it enables comparative genomics within this ancient Gondwanan family. The genus *Eucryphia* represents an interesting biogeographic story with disjunct distributions between eastern Australia, Tasmania and South America, reflecting ancient continental connections [24]. Understanding the genomic basis of its nectar production could have economic implications for the honey industry.
*Eupomatia laurina*	Native Guava or Bolwarra belongs to the ancient family Eupomatiaceae within Magnoliales, representing one of the earliest diverging angiosperm lineages. This species produces edible fruits traditionally consumed by Indigenous Australians, and the aromatic leaves were used for medicinal purposes [25]. As the first genome for this family, it provides crucial data for understanding early angiosperm evolution and the genetic basis of primitive floral characteristics. Its unique beetle-pollinated flowers lack true petals and represent an ancestral flower type.
*Flindersia xanthoxyla*	Yellow Teak is a large rainforest tree of Rutaceae, providing a second representative of this diverse family in this study. The genus *Flindersia* contains commercially important timber species used in furniture and construction, with some species threatened by habitat loss. Rutaceae includes many species with essential oils and bioactive compounds. The reference genome could assist in efforts to develop Greening Disease resistance [26].
*Gastrolobium racemosum*	The Net-Leaved Poison is a Western Australian endemic from Tribe Mirbelieae, a group comprising over 70% of Australian native legumes with significant ecological and evolutionary importance. Many *Gastrolobium* species produce fluoroacetate, the compound used in “1080” poison for pest control [27], and native Australian fauna have evolved resistance to this toxin, presenting a unique system for studying coevolutionary adaptation. This genus has undergone recent taxonomic revision and contains numerous rare and threatened species.
*Gymnostoma australianum*	A narrow endemic restricted to the Wet Tropics of northern Queensland and represents the earliest diverging lineage within Casuarinaceae, an ancient Gondwanan family [28]. As the first genome for this tropical genus, it enables comparison with published *Casuarina* genomes to investigate genomic changes underlying the shift from tropical rainforest ancestors to the arid-adapted sclerophyllous forms of *Casuarina* and *Allocasuarina*. Casuarinaceae are significant nitrogen-fixing plants in Australian ecosystems. Its restricted distribution makes it a species of conservation concern in the face of climate change.
*Hedycarya angustifolia*	Austral Mulberry is a member of Monimiaceae, a largely tropical family with significant representation in Australian rainforests. Monimiaceae produce diverse secondary metabolites including aromatic oils used in traditional medicine. The family has complex floral structures and unusual wood anatomy.
*Isopogon anethifolius*	The Proteaceae has an extensive diversification and radiation within southern hemisphere Mediterranean environments. While there are a number of genomic resources within the Grevilleioideae subfamily, until recently there were none in the other two subfamilies (Proteoideae, Persoonioideae). A recent genome for a South African *Protea* has been published [29] and this genome will add a second resource in the Proteoideae.
*Kingia australis*	*Kingia* (Dasypogonaceae) exhibits convergent evolution with *Xanthorrhoea* (grass trees). This Western Australian endemic represents an ancient lineage within the commelinid monocots, and its phylogenetic position has been debated [30].. The species has cultural importance to Noongar people, who used the pith as food [25].
*Lepidosperma gladiatum*	A Swordsedge from the Cyperaceae, this represents the first genome for this diverse genus, which contains over 100 species across Australia [31]. Sedgerushes are ecologically important in wetland and heathland communities, providing habitat structure and food resources for wildlife. *Lepidosperma* has undergone significant diversification in Australia.
*Leucochrysum albicans*	*Leucochrysum albicans* is a member of the paper daisy and cudweed tribe Gnaphalieae, which comprises approximately half of the native Australian Asteraceae. This is the first published genome of the Australasian clade of this tribe. The subspecies chosen for sequencing, *L. albicans* subsp. tricolor, is also of conservation concern and listed as ‘endangered’ in Victoria [32,33].
*Nothofagus cunninghamii*	Myrtle Beech is a cool temperate rainforest tree endemic to southeastern Australia. This species was historically widespread but is now restricted to fragmented refugia in Tasmania and Victoria, with populations threatened by myrtle rust and climate change [34]. As the sole Australian member of the Lophozonia subgenus, it provides a phylogenetic link for understanding *Nothofagus* biogeography and the evolutionary history of southern hemisphere temperate forests. Indigenous Tasmanian Aboriginal people traditionally used the bark for medicinal purposes [35], and the timber was valued for its durability in early colonial woodworking. This genome represents the basal family within Fagales and will support ongoing mycorrhizal research critical to understanding forest ecosystem dynamics.
*Pelargonium australe*	Native Stork’s Bill is a widespread Australian native pelargonium found across all mainland states. The species has traditional uses by Indigenous Australians, who applied the plant medicinally for treating wounds and skin conditions [36]. Unlike the commercially important horticultural pelargoniums, *P. australe* represents an Australian lineage that has adapted to dry, fire-prone environments and provides insight into the evolution of drought tolerance in Geraniaceae. While a reference genome already exists in NCBI, the highly fragmented 5.5kb N50 assembly misses large repetitive portions, and this updated genome, approximately 2 × larger, is consistent with current size estimates and enables more accurate study of genome evolution in this genus [37].
*Phebalium stellatum*	Starry *Phebalium* is a member of Rutaceae, a family containing many Australian endemic genera with significant horticultural and ecological value. The genus includes species adapted to diverse Australian habitats from rainforests to arid zones. Rutaceae are characterized by oil glands producing aromatic compounds, and some species are used in essential oil production [38].
*Phyllanthus gunnii*	Represents the first genome for Phyllanthaceae, a large pantropical family with significant Australian representation. The family is one of the largest flowering plant groups with high morphological diversity [39], including species used in traditional medicine. Australian species include both endemic taxa and elements with broader distributions. Note that the species has been recently reclassified to the Australian endemic genus *Lysiandra* [40]. Due to the species not yet being renamed in the Australian Plant Census, or the voucher specimens, we have used *Phyllanthus gunnii* in this paper.
*Pultenaea daphnoides*	Daphne Bush-pea is a member of the diverse Tribe Mirbelieae, an almost entirely Australian endemic radiation comprising over 750 species within the Fabaceae. With currently no available genomes published, the genus is one of the largest Australian legume genera with over 100 species, many of which are narrow endemics with conservation significance in southeastern Australia. *Pultenaea* exhibits diverse floral morphologies likely adapted to different pollinators.
*Quintinia fawkneri*	A narrow endemic of the wet tropics of northern Queensland and represents the first genome for the family Paracryphiaceae and the order Paracryphiales. The genus has a disjunct distribution across the southwest Pacific, with this species representing one of the Australian taxa in an otherwise primarily Malesian genus. Paracryphiaceae occupy an isolated phylogenetic position within the campanulid clade, a poorly understood lineage [41]. The restricted distribution of *Q. fawkneri* in upland rainforests makes it vulnerable to climate change and habitat disturbance, highlighting its conservation significance.
*Roepera similis*	The first genome for Zygophyllaceae, a family with global distribution in arid and semi-arid regions. The genus is an important component of Australian arid zone vegetation and shows interesting adaptations to salinity and drought. Zygophyllaceae include species with medicinal properties used by Indigenous peoples and in traditional medicine systems.
*Wahlenbergia ceracea*	Waxflower Bluebell is a member of Campanulaceae found in southeastern Australian montane and subalpine habitats. The genus has a southern hemisphere distribution pattern reflecting Gondwanan biogeography, with radiation in Australia. This species has conservation significance given its restricted high-elevation habitats that are vulnerable to climate change.

The reference genomes presented here provide a starting point for investigating genome evolution across the Australian flora. They will support comparative analyses of adaptation to aridity, fire, and nutrient limitation, including the gene families and metabolic pathways underlying these traits. Beyond genome evolution, they provide a reference to investigate genetic variation data necessary for population genomic studies with direct applications in conservation. More broadly, these resources integrate Australia’s plant diversity into large-scale global genomic efforts and provide a foundation for research across one of the world’s most distinctive floras.

## Materials and methods

### Sampling

Data from public sequence repositories: NCBI (GenBank, SRA, NCBI genomes); and PlaBiPd (https://www.plabipd.de/) were queried (up to and including December 2025) to identify taxonomic gaps in reference genomes (and genomic data generally) for Australian angiosperms. Genomic resource gaps across the Australian angiosperm phylogeny were identified and lineages represented in secure living collections were prioritised. During sampling, additional material was collected to generate voucher specimens for deposition in herbaria, and leaf tissue was preserved for future research [42–44]. Under-represented orders and families were targeted, with an emphasis on diploid genomes ≤ 2 Gb to enable high-quality assemblies, with selected exceptions for larger genomes in distinctive lineages (*Austrobaileya*, *Berberidopsis*, and *Doryanthes*).

Twenty-nine plant species were selected for genome sequencing and assembly. In instances where genome sizes were unknown, flow cytometry was carried out prior to DNA extraction and sequencing [45] to ensure that genomes were tractable and for determining the initial sequencing coverage needed. Where possible, plant species selected for sequencing were sourced from accessioned material cultivated within living collections such as botanic gardens or plant nurseries. Herbarium voucher specimens were made for all sampled plants and lodged in herbaria (Table 2.). Young, healthy leaf material from the study species was harvested from actively growing plants then stored in a sealed bag at 4℃ until processing was possible. All plant material originated from botanic garden herbaria or living collections. Fig 1 shows a simplified tree of plant orders (following the APG IV classification) illustrating the numbers of genomes publicly available on the NCBI genomes repository (https://www.ncbi.nlm.nih.gov/home/genomes/), and the taxonomic positions of the 29 genomes presented here.

**Table 2:** Plant genome samples. NSW = New South Wales; Qld = Queensland; WA = Western Australia, PNG = Papua New Guinea.

Species	Order	Family	Distribution	Voucher accession
*Archirhodomyrtus beckleri*	Myrtales	Myrtaceae	Central-E. NSW, NE. Qld.	MEL2529501A
*Austrobaileya scandens*	Austrobaileyales	Austrobaileyaceae	NE. Qld.	CNS153187
*Bellendena montana*	Proteales	Proteaceae	Tasmania	HO613866
*Berberidopsis beckleri*	Berberidopsidales	Berberidopsidaceae	NE. NSW, SE. Qld.	NE113249
*Callicoma serratifolia*	Oxalidales	Cunoniaceae	SE. Qld., E. NSW	MEL2529498A
*Dampiera purpurea*	Asterales	Goodeniaceae	E. Australia	MEL2525469A
*Dendrocnide excelsa*	Rosales	Urticaceae	E. Australia	MEL2060986A
*Denhamia bilocularis*	Celastrales	Celastraceae	E. Qld., NE. NSW	MEL2524932A
*Doryanthes excelsa*	Asparagales	Doryanthaceae	E. NSW	MEL2524933A
*Elaeocarpus reticulatus*	Oxalidales	Elaeocarpaceae	E. Australia	MEL2524938A
*Eremophila drummondii*	Lamiales	Scrophulariaceae	SW. WA	MELUD155122a
*Eucryphia lucida*	Oxalidales	Cunoniaceae	Tasmania	MEL2529901A
*Eupomatia laurina*	Magnoliales	Eupomatiaceae	E. Australia, PNG	MEL2524934A
*Flindersia xanthoxyla*	Sapindales	Rutaceae	NE. NSW, SE. Qld.	MEL2457406A
*Gastrolobium racemosum*	Fabales	Fabaceae	S. WA	MEL2529504A
*Gymnostoma australianum*	Fagales	Casuarinaceae	NE. Qld.	MEL2524930A
*Hedycarya angustifolia*	Laurales	Monimiaceae	SE. Australia	MEL2529902A
*Isopogon anethifolius*	Proteales	Proteaceae	E. NSW	MEL2524935A
*Kingia australis*	Arecales	Dasypogonaceae	SW. WA	KPBG30714
*Lepidosperma gladiatum*	Poales	Cyperaceae	S. Australia	MEL2529500A
*Leucochrysum albicans*	Asterales	Asteraceae	SE. Australia	CANB920543
*Nothofagus cunninghamii*	Fagales	Nothofagaceae	Tasmania and S. Vic.	MEL2524931A
*Pelargonium australe*	Geraniales	Geraniaceae	S. Australia	AD298752
*Phebalium stellatum*	Sapindales	Rutaceae	NE. NSW	NE109286
*Phyllanthus gunnii*	Malpighiales	Phyllanthaceae	E. Australia	MEL2529502A
*Pultenaea daphnoides*	Fabales	Fabaceae	SE. Australia	MEL2478484A
*Quintinia fawkneri*	Paracryphiales	Paracryphiaceae	NE. Qld.	MEL2529504A
*Roepera similis*	Zygophyllales	Zygophyllaceae	Australia	MEL2478506A
*Wahlenbergia ceracea*	Asterales	Campanulaceae	SE. Australia	CANB924602

**Fig 1:**
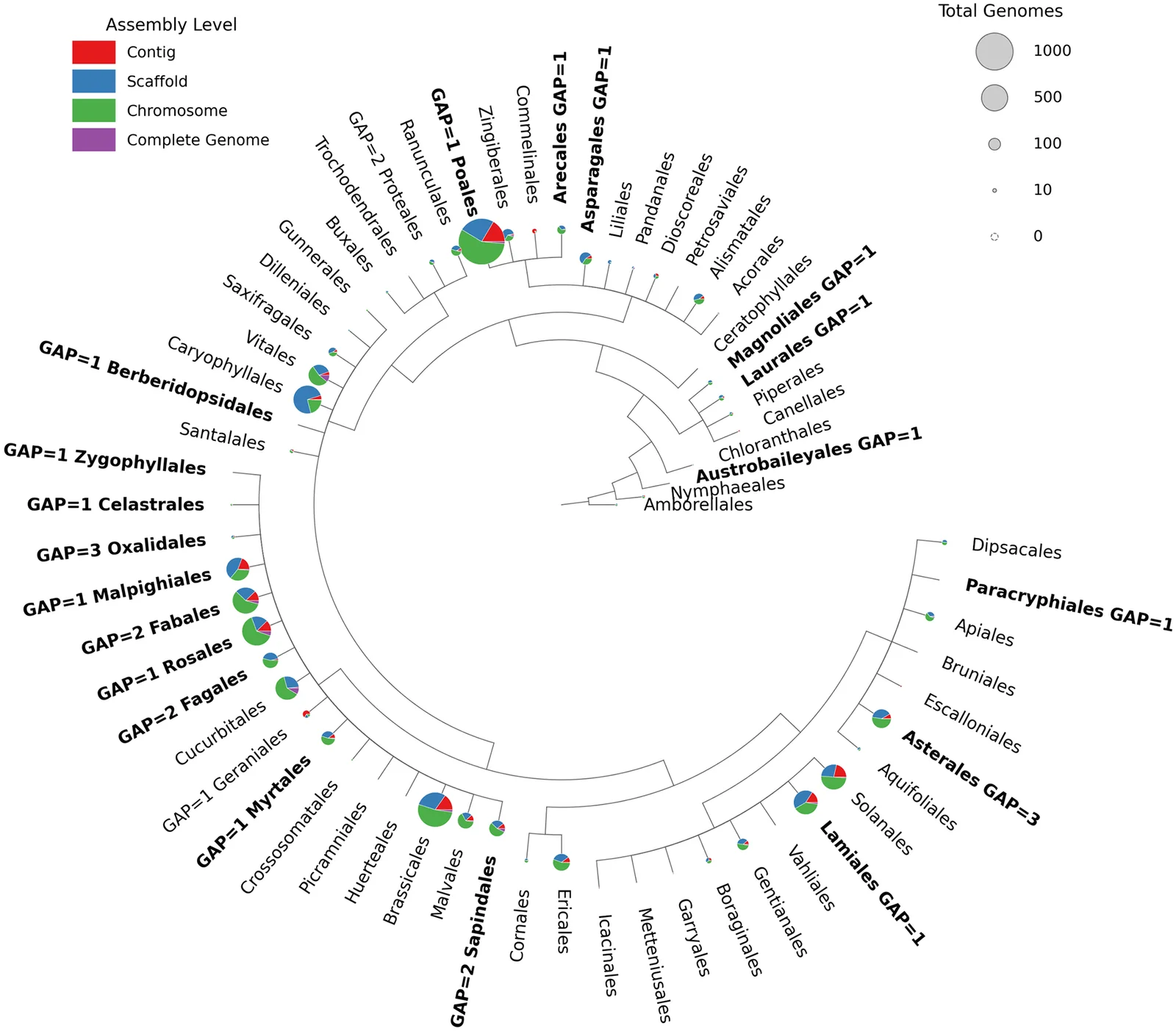
Simplified tree of APG IV 64 plant orders illustrating gaps in current genome availability. The total number of genomes published in the NCBI genomes repository is illustrated by pie charts for each order. The radii of the pie charts are linearly proportional to the total number of published genomes. Each segment shows the proportion of NCBI’s classification of genome completeness level, ascending = contig, scaffold, chromosome, complete genome. Orders shown in bold are those containing the current GAP genomes.

### High-molecular weight DNA extractions

High-molecular weight (HMW) DNA was extracted from all species, except those detailed below*,* using a magnetic bead-based method [46]. DNA was challenging to extract for *Archirhodomyrtus beckleri* and *Isopogon anethifolius*, with secondary metabolite and complex polysaccharide contaminants. These species were instead extracted with a cetrimonium bromide (CTAB) method. This method was originally developed for recalcitrant fungal samples, which is described elsewhere [47], and is now further refined in [48]. Briefly, cryogenically ground leaf tissue was lysed in high-salt CTAB buffer, followed by chloroform phase separation and selective DNA precipitation in low-salt CTAB to exclude co-precipitating secondary metabolites and polysaccharides as much as possible. *Eremophila drummondii* was separately extracted using a modified SDS-based method [12]. After HMW DNA extraction, some species benefitted from further rounds of DNA clean-up (*Isopogon anethifolius* and *Gastrolobium racemosum*), which is detailed in [49]. For species with sufficient HMW DNA yield and purity, PacBio HiFi long read sequencing was performed. However, HiFi required higher yield and purity of DNA than ONT (Oxford Nanopore Technology), therefore more recalcitrant species were sequenced using ONT. Larger and more complex genomes were sequenced using both technologies.

### PacBio HiFi long-read DNA sequencing

The HMW DNA was sheared to ~18 kb fragments on a Megaruptor 3 (Diagenode) using one cycle at 31x speed followed by two cycles at 32x speed. Sheared DNA was repaired with a 0.5x reaction of NEBNext FFPE DNA Repair Mix (New England BioLabs M6630) for 10 min at room temperature, then size-selected for fragments ≥15 kb on a BluePippin (Sage Science). PacBio single molecule, real-time (SMRT) libraries were prepared following the manufacturer’s SMRTbell Express Template Prep Kit 2.0 protocol (Pacific Biosciences). Sequencing was performed on a PacBio Sequel II with an 8M SMRT cell at Genomics WA (Perth, Australia) in circular consensus sequencing (CCS) mode to generate highly accurate HiFi reads (≥Q20). Species with larger genomes (*Austrobaileya scandens*, *Bellendena montana*, *Doryanthes excelsa*) were prepared with the SMRTbell Express Template Prep Kit 3.0 and sequenced on a PacBio Revio with a 25M SMRT cell at the Biomolecular Resource Facility, Australian National University (Canberra, Australia).

### Oxford Nanopore Technologies (ONT) long-read DNA sequencing

The HMW DNA was size-selected on a BluePippin (Sage Science) with a threshold of ≥20 kbp. Native DNA sequencing libraries were prepared following the manufacturer’s Ligation Sequencing DNA V14 protocol (SQK-LSK114) and sequenced on an Oxford Nanopore Technologies (ONT) PromethION with a FLO-PRO114M R10.4.1 flow cell at the Biomolecular Resource Facility, Australian National University (Canberra, Australia). When pore occupancy declined, the flow cell was washed and reloaded with fresh library following the manufacturer’s Flow Cell Wash Kit protocol (EXP-WSH004). The wash was performed at least twice to maximise sequencing output. Basecalling was performed with ONT Dorado v0.7.2 using the super-accurate model (DNA R10.4.1 e8.2 400bps sup).

### Bioinformatics

All scripts used to process reads, assemble, and analyse genomes are provided in the repository: https://github.com/Royal-Botanic-Gardens-Victoria/GAP_Reference_Genomes/. ONT sequence reads were filtered using chopper v.0.2.0 [50], (settings: length = 5000 bp; quality Q-value = 12; headcrop = 200; tailcrop = 200). PacBio HiFi reads were not filtered prior to steps below. ONT and HiFi reads were assessed for their expected genome coverage using GenomeScope v1.0.0 before assembly [51]. Where the read coverage fell below 12X, sequencing runs were replicated. Filtered ONT and HiFi reads were then mapped to plastid and mitochondrial NCBI RefSeq databases (all ‘viridiplantae’ RefSeq complete plastid and mitochondrial genomes, 24/6/2025) to remove organelle genome reads prior to nuclear genome assembly. This enabled the separate assembly of organelle genomes (see below). This was done because the HiFi assembler, hifiasm [52], preferentially removes high coverage contigs, which could cause the loss and / or poor assembly of organelle genomes. Nuclear plant genomes also contain nuclear insertions of plastid and mitochondrial sequences (NUMTs and NUPTs respectively; [53]), the assembly of which could be confounded and mis-directed by the presence of high copy number organelle-origin reads. See the scripts cp_bbmap.sh and mt_bbmap.sh for programmatic details.

Nuclear genomes were assembled with hifiasm v0.20.0-r639 [52] with the exception of *Callicoma serratifolia* which was assembled using canu v2.3 [54]. Program options are detailed in the script hifiasm.sh and canu.sh. ONT reads were corrected using the HERRO v.1 algorithm of the Dorado basecaller v0.7.2 (Oxford Nanopore Technologies PLC) and then assembled by hifiasm as per HiFi reads. However, *C. serratifolia* gave a low yield of reads (below 12X threshold) when passed through HERRO correction and was therefore assembled with canu because hifiasm is not suited for use with higher error-rate ONT reads (see script canu.sh).

Organelle genomes were assembled using hifiasm with an approximate 100X coverage sub-sample of reads that mapped to the plastid and mitochondrial RefSeq databases (based on mean expected plastid and mitochondrial genome sizes of 150 kbp and 500 kbp respectively), see scripts cphifiasm.sh and mthifiasm.sh. Plastomes were annotated using a local installation of Chloe (https://github.com/ian-small/Chloe.jl). Mitomes were annotated with Mfannot v1.37 [55] and rRNA genes annotated additionally using barrnap v0.9 (https://github.com/tseemann/barrnap) with a custom script, mfannot-folder.py.

Nuclear genome assemblies were purged of haplotigs and repetitive contigs using purge_haplotigs v1.1.2 [56], see scripts purge-hist.sh and purge.sh. For the purpose of purging, assembly repeats were annotated with the Ensembl scripts, Red2Ensembl.py and AnnotRedRepeats.py (https://github.com/Ensembl/plant-scripts [57],.

Assembly contiguity and size statistics N50, L50, N95, and L95 were calculated with a python script ‘n50folder.py’. Genome quality and completeness in terms of gene content were assessed using compleasm v0.2.2 [58] which uses the BUSCO lineage database, ‘embryophyta_odb10.2019-11-20’. Putative telomere repeats were identified using tidk v0.2.65 [59] – performed only on contigs > 1 Mbp in length (assumed as an approximate minimum chromosome size). Read quality and genome completeness were also assessed using Merqury v1.4.1 [60]. Merqury % completeness was calculated using combined haploid genomes obtained from hifiasm. We found that if the collapsed haploid genome was used, completeness was underestimated due to heterozygosity.

Telomere repeat motifs were not assumed prior to analysis. Initially, the most abundant repeat motif candidate identified by the ‘tidk explore’ script was used for ‘tidk search.’ If this sequence was not found to be a good candidate (few or no peaks in abundance at ends of contigs), then the next most abundant motif was searched for. This was repeated until repeat motif abundance was less than 10,000, at which point a result of ‘no telomeres found’ was recorded. Where no canonical telomere repeat motifs could be identified with TIDK, the script TelosearchLR.py [61] was used to check for presence of novel telomere repeats. Telomere repeat positions on contigs were recorded and classified as being at the start (5`), end (3`) or both ends of a contig based on their presence being in the first 10% or the last 10% of the total contig length. Genome ploidy was investigated with nQuire [62] – as recommended by the authors, the most likely ploidy between diploid, or tetraploid was chosen based on the model with the lowest ΔlogL value. The 40 largest assembled contigs (or fewer where fewer than 40 contigs were assembled) were tested with nQuire individually and where a clear consensus of ploidy was observed, that level was selected as the most likely ploidy.

Contigs of potentially bacterial or fungal contamination origin were identified using BLAST 2.11.0+ with RefSeq Genomes databases (bacteria, archaea, and fungi July, 2022). Python scripts ‘concat-blast.py’ and ‘contig-proportion.py’ were used to concatenate tandem blast hits and to quantify the proportion of bacterial or fungal sequence in each contig. Contigs with more than 10% bacterial or fungal sequence were removed. All removed contigs were small (less than 50 kbp).

Nuclear genomes were annotated with the GeMoMa v1.9 [63] GeMoMaPipeline command (200 kb maximum intron size). Eight plant NCBI RefSeq genomes were used as annotation references representing a diverse spread of taxa: *Eucalyptus grandis* GCF_016545825.1, *Humulus lupulus* GCF_963169125.1, *Oryza sativa* GCF_034140825.1, *Pisum sativum* GCF_024323335.1, *Quercus robur* GCF_932294415.1, *Salvia miltiorrhiza* GCF_028751815.1, *Telopea speciosissima* GCF_018873765.1, and *Tripterygium wilfordii* GCF_013401445.1. Three annotations failed due to errors associated with genes from one of the reference genomes. These were re-run without the offending reference: no *H. lupulus* reference for *Phebalium stellatum* and *Pultenaea daphnoides;* and no *Quercus robur* reference for *Roepera similis.* A repeat database was created for each genome using RepeatModeler v2.0.4, and repeats were classified and reported using RepeatMasker v4.2.0 (http://www.repeatmasker.org). Note that genomes were not repeat-masked prior to annotation due to the GeMoMa pipeline being entirely reference based. Annotation gff3 and fasta files are available online, https://zenodo.org/records/22038808.

## Results and discussion

The long-read sequencing technology, HiFi, and HERRO corrected ONT, combined with the assembler hifiasm (or Canu when ONT read correction could not be performed) was successful in producing high quality, contiguous reference genomes suitable for a wide range of genomic research. In addition, organelle read filtering and separate organelle assembly, provided reference organelle genomes for all species except the *Pelargonium australe* mitochondrion. The bioinformatics pipeline detailed here was relatively straightforward, and assemblies did not require ‘polishing’ correction or downstream scaffolding steps due to the low error rate expected from HiFi technology and/or HERRO correction. We have not attempted to assemble or report the haplotypes of putative polyploids in this work. While hifiasm with long read data is capable of attempting to phase haplomes, in the absence of trio samples (parents and offspring of controlled crosses) or Hi-C topological linking, the accurate production of haplomes is not possible. The details of the assemblies below therefore refer to only *purged* haploid genomes, in which likely homeologs have been removed. GenomeScope (see supplementary file S1 File) was a useful tool for decision making in the sequencing and assembly process. GenomeScope produced histograms of kmer frequencies together with expected models for the estimated genome size, when histograms in genomescope plots were sparsely populated, or model fitting failed, this clearly indicated when sequencing depth was insufficient, in which case further HiFi or ONT sequencing was performed.

Sequence reads and genome assemblies were deposited in the NCBI Genomes repository (https://www.ncbi.nlm.nih.gov/home/genomes/) under study accession PRJEB88028. Genome assemblies are also available in the repository: https://zenodo.org/records/22038808. Estimated genome sizes and assembly statistics are shown in Table 3. Read coverage after filtering was high (28x - 175x) – well above the minimum recommended for assembly by hifiasm and Canu authors (13x and 20x respectively). Previously estimated haploid genome lengths and observed haploid assembly lengths had a wide range (0.3–9 Gbp estimated; 0.24–7.9 Gbp observed). The expected genome lengths and observed assembly length were closely correlated (Fig 2), R^2^ = 0.9122, with the mean observed length approximately 20% lower than the flow cytometry based estimates (correlation gradient = 0.8). Notably, the observed genome size of *Kingia australis* was 2.9 times the expected size (estimated from *Dasypogon* in the same family in the c-values database, https://cvalues.science.kew.org/). Pairwise dot plots of the 20 largest contigs from the assembly did not reveal any visible syntenic blocks (see supplementary file, S1 Fig) that could indicate unpurged haplotigs or homoeologs in the assembly.

**Table 3:** Genome assembly statistics of purged assemblies. All species were assembled using hifiasm, except *Callicoma serratifolia* which was assembled using Canu. HERRO ONT (Oxford Nanopore Technologies) reads were corrected using the HERRO algorithm. “HiFi’ = PacBio HiFi reads. Haploid genome size estimates were made prior to sequencing using flow cytometry (* or existing literature estimates).

Species	Sequencing method	Est. haploid size (Gbp)	Exp. coverage	Obs. coverage	Contigs	Length (Gbp)	N50	L50	N95	L95
*Archirhodomyrtus beckleri*	HiFi + ONT (HERRO)	0.8*	225	175	28	0.40	3.17E + 07	6	1.03E + 07	13
*Austrobaileya scandens*	HiFi + ONT	9.3*	56	64	454	7.93	4.45E + 07	44	5.70E + 06	259
*Bellendena montana*	HiFi + ONT (HERRO)	4.96*	46	28	8151	4.52	2.48E + 06	506	1.00E + 05	4361
*Berberidopsis beckleri*	HiFi	0.3*	206	57	389	0.72	1.31E + 07	18	1.27E + 06	77
*Callicoma serratifolia*	ONT	Unknown	N/A	104	544	0.28	9.58E + 05	90	1.76E + 05	359
*Dampiera purpurea*	HiFi	0.5*	80	18	448	1.37	8.03E + 06	48	9.76E + 05	226
*Dendrocnide excelsa*	HiFi	1.6	90	54	232	0.92	1.43E + 07	24	2.76E + 06	74
*Denhamia bilocularis*	HiFi	0.8	72	137	108	0.29	1.94E + 07	6	4.21E + 06	15
*Doryanthes excelsa*	HiFi + ONT	3.2*	27	32	64	2.60	8.63E + 07	11	3.64E + 07	30
*Elaeocarpus reticulatus*	HiFi	0.3	49	45	58	0.32	1.75E + 07	8	4.43E + 06	21
*Eremophila drummondii*	HiFi	0.8	58	40	55	1.14	3.35E + 07	13	1.55E + 07	33
*Eucryphia lucida*	ONT (HERRO)	Unknown	N/A	61	29	0.31	1.80E + 07	8	8.42E + 06	17
*Eupomatia laurina*	HiFi	1.2*	50	62	34	0.94	8.11E + 07	6	2.19E + 07	13
*Flindersia xanthoxyla*	HiFi	0.4	103	97	68	0.40	1.45E + 07	11	4.82E + 06	29
*Gastrolobium racemosum*	ONT (HERRO)	0.85	80	38	484	1.12	4.45E + 06	87	1.18E + 06	273
*Gymnostoma australianum*	HiFi	0.7*	68	133	35	0.31	3.38E + 07	4	3.84E + 06	12
*Hedycarya angustifolia*	HiFi	0.7	39	42	56	0.65	2.67E + 07	10	5.33E + 06	28
*Isopogon anethifolius*	ONT (HERRO)	0.6*	63	72	29	0.51	3.25E + 07	7	1.59E + 07	17
*Kingia australis*	HiFi	0.5*	134	45	246	1.47	1.37E + 07	36	2.64E + 06	137
*Lepidosperma gladiatum*	HiFi	0.6*	57	137	95	0.24	4.35E + 06	23	2.37E + 06	54
*Leucochrysum albicans*	HiFi	0.9	76	31	283	1.94	1.19E + 07	47	2.51E + 06	198
*Nothofagus cunninghamii*	HiFi	1*	34	52	76	0.54	2.72E + 07	8	8.70E + 06	21
*Pelargonium australe*	HiFi	1*	76	159	88	0.43	2.18E + 07	8	5.80E + 06	24
*Phebalium stellatum*	HiFi + ONT	1.5*	54	117	54	0.59	3.28E + 07	8	1.41E + 07	19
*Phyllanthus gunnii*	HiFi	1	81	81	103	0.95	2.14E + 07	16	5.30E + 06	52
*Pultenaea daphnoides*	HiFi	0.7*	47	56	28	0.58	5.04E + 07	6	2.10E + 07	13
*Quintinia fawkneri*	HiFi + ONT	1.2	97	101	127	1.12	3.09E + 07	14	5.67E + 06	44
*Roepera similis*	HiFi	1.2*	47	55	174	0.90	2.86E + 07	10	8.40E + 06	34
*Wahlenbergia ceracea*	HiFi + ONT (HERRO)	0.8*	52	38	932	0.78	1.41E + 06	161	2.93E + 05	634

**Fig 2:**
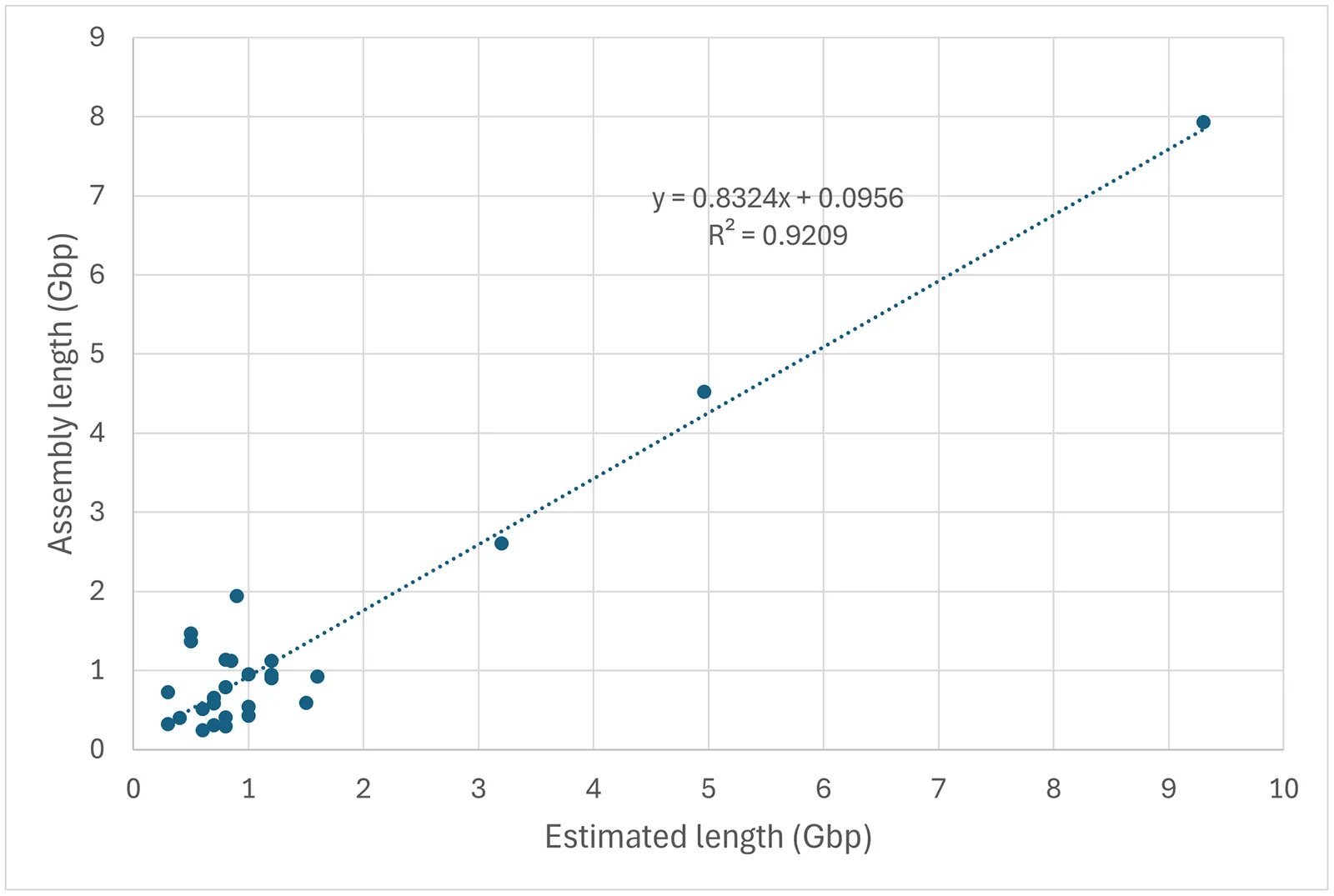
Assembled genome length (purged assembly) Vs. estimate of genome size.

The contiguity of assemblies was high, based on the generally low number of contigs (L95 range = 12–1784, mean = 152; N50 range = 9.6 x 10^5^–8.7 x 10^7^, mean = 2.8 x 10^7^). Many contigs were likely to represent complete telomere to telomere (T2T) chromosomes, as shown by the TIDK results (Table 4) – 17 assemblies had T2T contigs, and 29 genomes had contigs with at least one telomere. Telomere recovery was very variable among assemblies. The *Denhamia bilocularis* assembly had the highest proportion of T2T contigs (86% of the total assembly length, where the assembly was filtered to contigs > 1 Mbp), whereas in 12 species, no T2T contigs were assembled. All identified telomeres were the canonical *Arabidopsis* type telomere repeat, (TTTAGGG)n. However in two assemblies, *Austrobaileya scandens* and *Bellendena montana*, TIDK did not identify the repeat above the frequency threshold (n >= 10,000). Using contigs as input, TelosearchLR.py identified the presence of the repeat (ranking 5th and 4th respectively). It is possible that the prevalence of microsatellite repeats near contig ends prevented TIDK from identifying the canonical repeat.

**Table 4:** Counts of contigs with telomere repeat region candidates as detected by TIDK in contigs filtered to >1 Mbp in length; and % of assembly found within telomere-to-telomere (T2T) contigs. All telomere repeats were the Arabidopsis-type (TTTAGGG)n.

		Contig counts with telomere repeats				
Species	Contigs > 1Mbp	None	5’ only	3’ only	5’ and 3’	% T2T contigs
*Archirhodomyrtus beckleri*	14	0	5	3	6	56
*Austrobaileya scandens*	387	356	14	17	0	n/a
*Bellendena montana*	1145	1109	17	19	0	n/a
*Berberidopsis beckleri*	80	42	22	16	0	n/a
*Callicoma serratifolia*	82	79	2	1	0	n/a
*Dampiera purpurea*	224	214	5	5	0	n/a
*Dendrocnide excelsa*	90	64	12	14	0	n/a
*Denhamia bilocularis*	17	2	1	2	12	86
*Doryanthes excelsa*	36	3	10	9	14	54
*Elaeocarpus reticulatus*	22	2	10	5	4	27
*Eremophila drummondii*	38	6	14	16	2	12
*Eucryphia lucida*	21	2	7	3	9	58
*Eupomatia laurina*	18	2	7	7	2	17
*Flindersia xanthoxyla*	33	3	12	12	6	31
*Gastrolobium racemosum*	285	279	2	4	0	n/a
*Gymnostoma australianum*	17	4	9	1	3	34
*Hedycarya angustifolia*	36	11	10	7	8	40
*Isopogon anethifolius*	19	5	7	6	1	6
*Kingia australis*	173	163	7	3	0	n/a
*Lepidosperma gladiatum*	58	1	12	5	40	76
*Leucochrysum albicans*	247	219	14	14	0	n/a
*Nothofagus cunninghamii*	26	8	6	6	6	42
*Pelargonium australe*	29	15	9	5	0	n/a
*Phebalium stellatum*	21	1	5	7	8	48
*Phyllanthus gunnii*	66	48	5	13	0	n/a
*Pultenaea daphnoides*	15	1	4	9	1	9
*Quintinia fawkneri*	55	17	14	13	11	32
*Roepera similis*	44	11	10	11	12	36
*Wahlenbergia ceracea*	266	258	2	6	0	n/a

The completeness of assemblies, as measured using BUSCO genes, was generally high, ranging from 99.88% for *Elaeocarpus reticulatus* to 93.12% for *Dendrocnide excelsa* (Table 5). High gene duplication rates were present in some assemblies, notably, *Leucochrysum albicans* and *Eremophila drummondii*¸ (61.5% and 48.8% respectively). Merqury gave similarly high completeness (82.2% − 99.6%) and high QV (39.84–71.79) (Table 5). Merqury % completeness and QV could not be calculated for *C. serratifolia* because in our pipeline Canu only output one haplotype, which causes Merqury to underestimate. *D excelsa* stands out as having the lowest completeness in BUSCO (93.12%) and Merqury (82.2%). The reason for this is currently unknown, and the completeness of its assembly may benefit from further sequencing with, e.g., Hi-C methods. Results of per contig ploidy tests as performed by nQuire are shown in Table 6, and detailed per contig results are shown in S2 File. Assemblies where over half of tested contigs were designated tetraploid were assigned as putative tetraploids: 13 genomes, ranging from 55% (*Wahlenbergia ceracea*) to 100% tetraploid contigs (*Bellendena montana* and *Dendrocnide excelsa* – note that *Bellendena* also had four triploid contigs according to nQuire); and 11 of the remaining assemblies were designated as putative diploid genomes, ranging from 3% (*Hedycarya angustifolia*) to 30% tetraploid contigs (*Nothofagus cunninghamii*). There was no clear threshold between these designations, and it is likely that intermediate proportions of ploidy represent genomes in the process of rediploidisation [64]. When viewed in combination with the proportion of BUSCO duplicated genes, the proportion of tetraploid contigs generally increases with BUSCO duplication with a strong logarithmic correlation (Fig 3. R^2^ = 0.77). However, two groups are present above and below the general trend: first, five genomes with a high proportion of tetraploid contigs and low BUSCO duplication (high C/B ratio): *Gymnostoma australianum*, *Berberidopsis beckleri*, *Austrobaileya scandens*, *Roepera similis*, and *Phebalium stellatum*; and second, two genomes with a low proportion of tetraploid contigs and high BUSCO duplication (low C/B ratio): *Flindersia xanthoxyla*, and *Eremophila drummonii*. The first group could be interpreted as a stable autotetraploid group where BUSCO genes are in lower copy numbers throughout following homeolog purging of the assembly. The second group could be interpreted as alloploids or aneuploids with BUSCO genes more frequently duplicated, possibly in the process of rediploidisation. However, among the large diversity of angiosperm genomes sequenced here, many other factors could confound this interpretation, including repeat content, mating system, age of the polyploidisation event, and unknown assembly errors. Complete phasing of genomes using HiC linkage or trio sequencing could resolve these unknowns.

**Table 5:** Assembled purged genome completeness assessed using compleasm and Merqury. Values are the observed % of genes found from the BUSCO embryophyta database (https://busco-data.ezlab.org/v4/data/lineages/embryophyta_odb10.2019-11-20.tar.gz). S = single copy complete genes; D = duplicated genes; F = fragmented genes; M = missing genes. QV = Merqury per base quality value; %Comp.  = Merqury percentage completeness.

Species	All	S	D	F	M	QV	%Comp.
*Archirhodomyrtus beckleri*	99.13	96.65	2.48	0.5	0.37	61.98	99.2794
*Austrobaileya scandens*	98.89	91.76	7.13	0.5	0.62	49.18	98.8283
*Bellendena montana*	99.44	69.7	29.74	0.25	0.31	55.06	99.6
*Berberidopsis beckleri*	98.82	93.93	4.89	0.37	0.81	59.75	99.0439
*Callicoma serratifolia*	99.62	94.42	5.2	0.25	0.12	42.47	N/A
*Dampiera purpurea*	98.82	82.03	16.79	0.5	0.68	64.66	96.7489
*Dendrocnide excelsa*	93.12	59.6	33.52	1.86	5.02	65.01	82.2043
*Denhamia bilocularis*	99.19	93.43	5.76	0.56	0.25	66.81	99.1924
*Doryanthes excelsa*	99.5	76.95	22.55	0.37	0.12	63.65	99.4305
*Elaeocarpus reticulatus*	99.88	97.96	1.92	0.12	0	65.70	98.4451
*Eremophila drummondii*	99.25	55.45	43.8	0.19	0.56	71.79	98.3878
*Eucryphia lucida*	99.07	95.91	3.16	0.25	0.68	61.49	97.578
*Eupomatia laurina*	99.19	97.21	1.98	0.43	0.37	69.14	98.4455
*Flindersia xanthoxyla*	97.95	78.31	19.64	0.62	1.43	68.78	99.3351
*Gastrolobium racemosum*	99.57	60.04	39.53	0.37	0.06	51.71	99.1185
*Gymnostoma australianum*	99.82	98.27	1.55	0.19	0	68.01	99.5568
*Hedycarya angustifolia*	99.44	96.84	2.6	0.31	0.25	59.65	98.847
*Isopogon anethifolius*	97.4	88.66	8.74	0.31	2.29	64.79	96.347
*Kingia australis*	99.01	96.47	2.54	0.74	0.25	63.45	98.0141
*Lepidosperma gladiatum*	96.29	95.11	1.18	1.3	2.42	68.09	99.039
*Leucochrysum albicans*	98.88	37.36	61.52	0.19	0.93	64.68	97.5239
*Nothofagus cunninghamii*	99.81	95.6	4.21	0.19	0	65.66	98.9955
*Pelargonium australe*	95.54	87.42	8.12	1.05	3.4	61.79	98.316
*Phebalium stellatum*	98.94	91.57	7.37	0.25	0.81	54.80	96.2917
*Phyllanthus gunnii*	98.7	96.9	1.8	0.5	0.81	66.30	98.3503
*Pultenaea daphnoides*	99.69	96.41	3.28	0.31	0	65.65	98.2953
*Quintinia fawkneri*	99.5	95.16	4.34	0.19	0.31	55.11	98.9251
*Roepera similis*	98.46	91.33	7.13	0.43	1.12	67.62	98.6584
*Wahlenbergia ceracea*	97.58	83.95	13.63	0.43	1.98	39.84	94.3319

**Table 6:** Nquire tests of assembled genome ploidy, showing counts of contigs and their most likely ploidy (lowest DlogL value), where the 40 largest contigs were modelled separately (except in cases when fewer than 40 contigs were assembled). ‘*’ indicates uncertain ploidy / rediploidisation.

Species	Total contigs tested	% of genome tested	Diploid	Triploid	Tetraploid	Most likely ploidy
*Archirhodomyrtus beckleri*	14	100.00	11	2	1	2
*Austrobaileya scandens*	40	47.94	0	10	30	4
*Bellendena montana*	40	9.08	0	4	36	4
*Berberidopsis beckleri*	40	77.79	7	6	27	4
*Callicoma serratifolia*	40	29.15	31	4	5	2
*Dampiera purpurea*	40	44.78	3	9	28	4
*Dendrocnide excelsa*	40	72.65	0	0	40	4
*Denhamia bilocularis*	17	98.34	14	1	2	2
*Doryanthes excelsa*	36	99.87	7	1	28	4
*Elaeocarpus reticulatus*	18	99.60	14	0	4	2
*Eremophila drummondii*	37	99.89	13	14	10	2*
*Eucryphia lucida*	21	100.00	10	4	7	2*
*Eupomatia laurina*	18	100.00	14	1	3	2
*Flindersia xanthoxyla*	33	99.34	24	3	6	2
*Gastrolobium racemosum*	40	28.70	1	4	35	4
*Gymnostoma australianum*	17	100.00	3	1	13	4
*Hedycarya angustifolia*	36	99.84	34	1	1	2
*Isopogon anethifolius*	19	100.00	16	0	3	2
*Kingia australis*	40	54.09	27	8	5	2
*Lepidosperma gladiatum*	40	78.01	35	1	4	2
*Leucochrysum albicans*	40	46.08	0	4	36	4
*Nothofagus cunninghamii*	26	99.65	17	1	8	2
*Pelargonium australe*	29	99.38	7	4	18	4
*Phebalium stellatum*	21	99.83	2	4	15	4
*Phyllanthus gunnii*	40	87.06	18	8	14	2*
*Pultenaea daphnoides*	14	100.00	13	0	1	2
*Quintinia fawkneri*	40	93.12	19	4	17	2*
*Roepera similis*	40	98.46	7	4	29	4
*Wahlenbergia ceracea*	40	21.52	11	7	22	4

**Fig 3:**
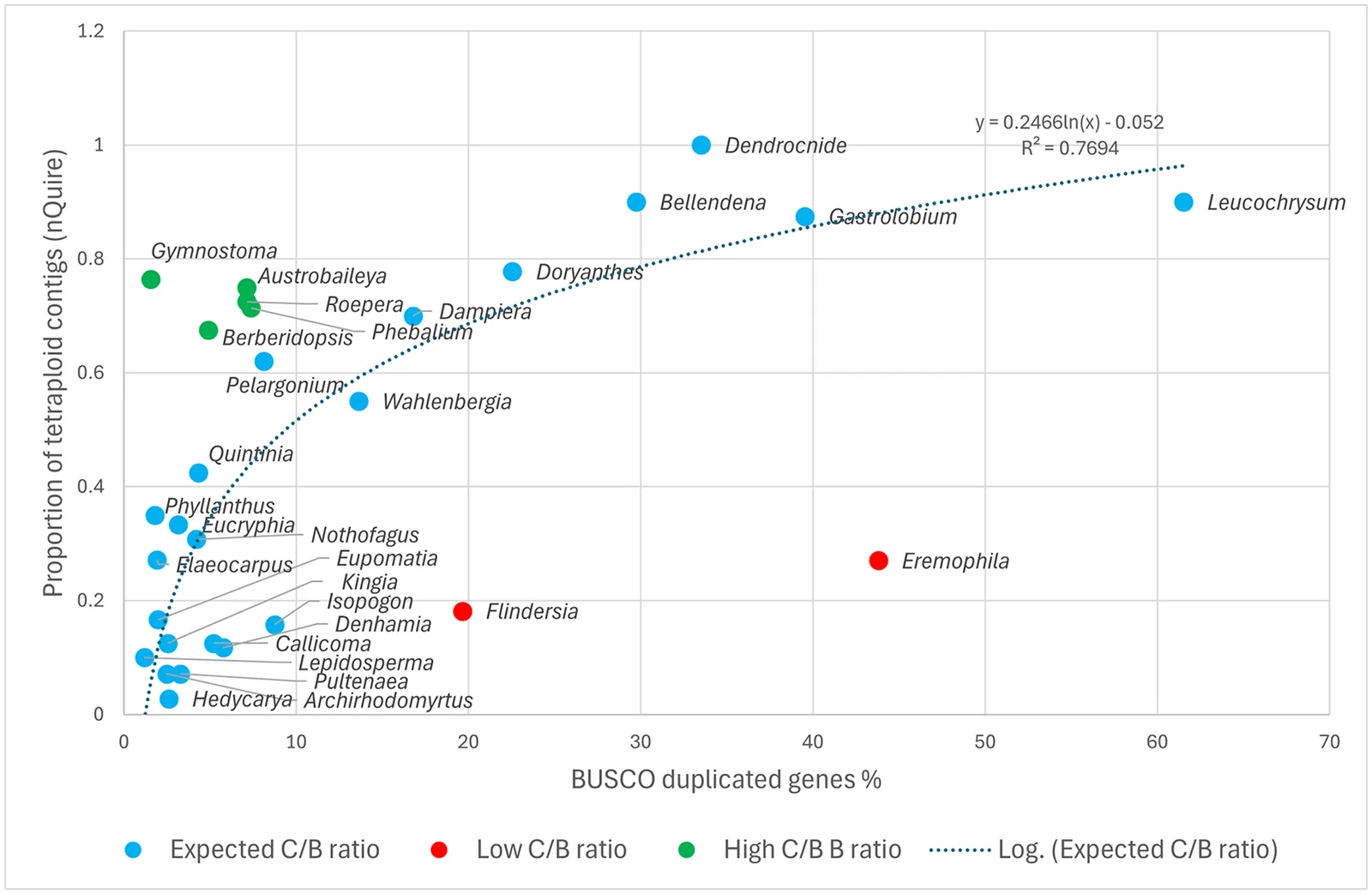
Proportion of tetraploid contigs (nQuire) Vs. % of duplicated genes (BUSCO). Genomes with higher than expected tetraploid contigs proportion to BUSCO gene duplication % (high C/B ratio) are marked in green. Genomes with low C/B ratio are marked in red.

The repeat content of each genome assembly as classified by RepeatModeler and RepeatMasker is summarised in Table 7. Total interspersed repeats comprised between 30.71% for *Callicoma serratifolia*, to 84.28% for *Austrobaileya scandens*. This is within the range expected for diverse plant genomes [65]. Repeat content was highly correlated to genome size with a log curve relationship (Fig 4), R^2^ = 0.8. The genome of *Phyllanthus gunnii* (*Lysiandra gunnii*) exhibited a higher than expected repeat content for its size (80.47%) as can be seen in its departure from the fitted curve in Fig 4. *Phyllanthaceae* are known to have high repeat content and variable ploidy [66]. Retroelements made up the largest proportion of classified repeats (4.18% − 45.45%).

**Table 7:** Assembled genome repeat content. Values are % of genome. Major plant repeats as annotated by RepeatModeller and RepeatMasker.

			**Retroelements**					
**Species**	**Total interspersed repeats**	**Simple repeats**	**Total**	**SINEs**	**Penelope**	**LINEs**	**RTE/Bov-B**	**L1/CIN4**
*Archirhodomyrtus beckleri*	49.09	0.82	20.66	0.38	0.01	1.46	0.08	1.34
*Austrobaileya scandens*	84.28	0.8	44.39	0.91	0	2.41	0.36	2.01
*Bellendena montana*	81.19	0.63	40.24	3.14	0.09	1.63	0.34	1.27
*Berberidopsis beckleri*	59.95	1	15.88	0.47	0.25	3.12	0.96	2.13
*Callicoma serratifolia*	30.71	1.52	6.57	0.7	0	1.18	0.14	0.99
*Dampiera purpurea*	72.77	1.08	32.69	0.03	0.01	1.46	0.19	1.27
*Dendrocnide excelsa*	71.47	1.28	33.32	0.27	0.07	0.28	0.01	0.25
*Denhamia bilocularis*	38.73	0.9	11.84	0.05	0.02	1.26	0.24	0.96
*Doryanthes excelsa*	72.93	1.58	36.96	0.3	0.36	1.69	0.79	0.86
*Elaeocarpus reticulatus*	38.67	1.43	7.22	0.01	0	1.07	0.09	0.99
*Eremophila drummondii*	71.16	0.57	27.32	0.02	0.05	1.29	0.07	1.23
*Eucryphia lucida*	38.19	1.68	11.3	0.33	0	1.82	0.08	1.73
*Eupomatia laurina*	61.39	1.34	14.81	0.57	0	2.58	1.12	1.45
*Flindersia xanthoxyla*	43.01	1.28	19.65	0.05	0	0.93	0.02	0.78
*Gastrolobium racemosum*	63.73	1.05	27	0.17	0.16	2.05	0.13	1.92
*Gymnostoma australianum*	36.03	0.81	14.34	0.36	0.01	2.32	0	2.21
*Hedycarya angustifolia*	58.83	1.32	14.66	0.94	0.14	3.01	0.71	2.21
*Isopogon anethifolius*	49.85	0.98	14.25	0.49	0.27	2.41	0.87	1.39
*Kingia australis*	68.94	1.03	41.9	2.26	0.26	1.17	0.15	0.97
*Lepidosperma gladiatum*	37.75	1.95	4.18	0.12	0	1.77	0.53	1.23
*Leucochrysum albicans*	73.96	1.27	40.24	0.19	0.11	1.31	0	1.29
*Nothofagus cunninghamii*	50.31	1.53	12.93	0.58	0.02	2.5	0.01	2.48
*Pelargonium australe*	53.14	1.34	20.46	0.47	0	2.53	0.08	2.4
*Phebalium stellatum*	63.4	1.35	25.61	0.12	0.28	1.16	0.04	1.12
*Phyllanthus gunnii*	80.47	0.57	45.45	0.12	0.01	0.77	0.01	0.75
*Pultenaea daphnoides*	58.39	1.05	23.49	0.11	0.16	0.95	0.17	0.66
*Quintinia fawkneri*	68.22	1.28	20.9	0.46	0.12	1.79	0.28	1.5
*Roepera similis*	68.47	0.87	26.4	0.06	0.02	4.29	2.18	2.1
*Wahlenbergia ceracea*	68.12	2.26	26.52	0.6	0.01	1.17	0.03	0.38
	**Retroelements** **continued**				**DNA transposons**			
**Species**	**LTR_elements**	**Ty1/Copia**	**Gypsy/DIRS1**	**Retroviral**	**Total**	**hobo- Activator**	**MULE-MuDR**	**Tourist/ Harbinger**
*Archirhodomyrtus beckleri*	18.83	8.21	9.85	0.4	1.27	0.6	0.51	0.08
*Austrobaileya scandens*	41.07	35.72	5.12	0.16	1.87	0.52	0.63	0.13
*Bellendena montana*	35.47	19.53	15.16	0.57	3.9	2.77	0.24	0.19
*Berberidopsis beckleri*	12.29	4.04	7.93	0.1	2.04	0.5	0.77	0.13
*Callicoma serratifolia*	4.69	1.48	2.92	0.09	1.92	0.69	0.54	0.18
*Dampiera purpurea*	31.19	19.69	11.23	0.09	0.78	0.15	0.42	0.04
*Dendrocnide excelsa*	32.76	16.81	15.84	0.1	3.39	0.84	0.42	0.07
*Denhamia bilocularis*	10.53	3.65	6.35	0.1	3.84	1.08	1.93	0.51
*Doryanthes excelsa*	34.98	8.49	24.47	1.36	5.34	0.59	1.04	0.08
*Elaeocarpus reticulatus*	6.14	2.18	3.1	0	1.32	0.48	0.48	0.1
*Eremophila drummondii*	26	8.96	16.58	0.14	5.96	0.51	0.76	1.66
*Eucryphia lucida*	9.15	0.65	8	0.07	1.46	0.6	0.39	0.17
*Eupomatia laurina*	11.66	6	4.88	0.29	4.3	0.8	0.99	0.29
*Flindersia xanthoxyla*	18.67	11.85	5.7	0.17	0.66	0.12	0.35	0.03
*Gastrolobium racemosum*	24.78	6.97	17.56	0.09	1.93	0.8	0.7	0.15
*Gymnostoma australianum*	11.66	8.64	2.59	0.04	0.18	0.08	0	0
*Hedycarya angustifolia*	10.72	5.98	3.61	0.4	4.1	1.85	0.52	0.08
*Isopogon anethifolius*	11.35	3.75	6.48	0.96	1.71	0.82	0.16	0.12
*Kingia australis*	38.47	13.18	23.07	1.69	2.55	0.8	0.51	0.42
*Lepidosperma gladiatum*	2.29	1.74	0.33	0.06	0.69	0.04	0.11	0.03
*Leucochrysum albicans*	38.74	11.99	26.58	0.01	2.79	0.29	0.48	0.37
*Nothofagus cunninghamii*	9.85	1.29	8.03	0.3	3.55	0.45	1.78	0.09
*Pelargonium australe*	17.46	2.83	14.28	0.15	2.25	0.4	0.42	0.2
*Phebalium stellatum*	24.33	9.69	14.35	0.01	3.57	1.11	0.65	0.59
*Phyllanthus gunnii*	44.55	28.65	14.97	0.5	4.07	0.41	0.74	0.15
*Pultenaea daphnoides*	22.44	7.08	14.57	0.39	1.73	0.55	0.54	0.1
*Quintinia fawkneri*	18.66	2.86	15.24	0.35	4.03	0.47	1.58	0.12
*Roepera similis*	22.06	17.5	4.34	0.12	2.72	1.33	0.27	0.14
*Wahlenbergia ceracea*	24.76	11.05	13.22	0.34	4.15	0.22	1.41	1.51

**Fig 4:**
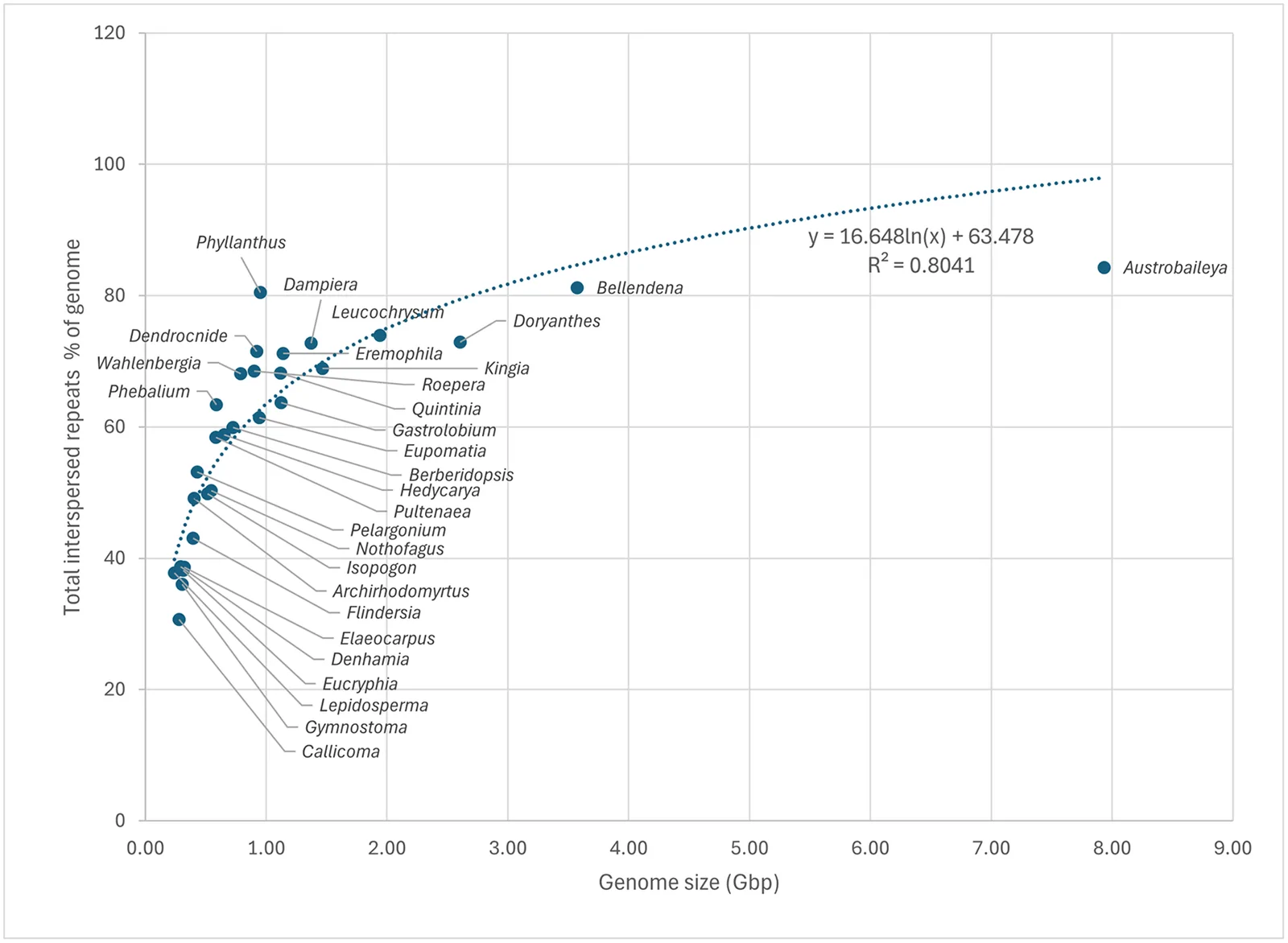
Total interspersed repeat content (% of genome) Vs. haploid genome assembly size.

The numbers of GeMoMA annotated genes per genome varied considerably: 24,712 in *Lepidosperma gladiatum,* to 91,604 in *Bellendena montana* (Table 8). A previous estimate of the number of genes in plants is c. 20,000–50,000 genes [1], however, five genomes presented here had counts over 70,000, which are unusually high. Of these genomes, all were likely tetraploids or in the process of rediploidisation (in the case of *E. drummondii*), which could account for the higher than expected gene counts. The number of genes was strongly correlated to genome size with a log relationship (Fig 5. R^2^ = 0.77), excluding *Austrobaileya scandens,* the largest genome, with c. 60% of expected genes given the underlying trend. Mean gene length (including introns) was also strongly log correlated to genome size (mean gene length (bp) = 3856 x ln(genome size (Gbp)) + 11136; R^2^ = 0.68), with *Callicoma seratifolia* having the smallest (5311 bp) and *Austrobaileya scandens* having the longest mean gene length (26,269 bp). It is therefore possible that the exceptionally long mean length of *Austrobaileya scandens* genes and high repeat content (see above) has limited its gene capacity compared to its large genome size.

**Table 8:** Genome assembly gene annotation (GeMoMa) details.

Species	Number of genes	Mean exons per cds	Mean gene length (bp)	Mean intron length (bp)
*Archirhodomyrtus beckleri*	42500	4.9	8530	1488
*Austrobaileya scandens*	59272	4.4	26272	8622
*Bellendena montana*	91604	4.2	12173	3342
*Berberidopsis beckleri*	40908	4.9	11515	2402
*Callicoma serratifolia*	33632	5.3	5314	820
*Dampiera purpurea*	54284	4.4	10041	2210
*Dendrocnide excelsa*	54116	5.0	10210	1719
*Denhamia bilocularis*	40402	5.2	6904	1057
*Doryanthes excelsa*	70532	4.5	14632	3896
*Elaeocarpus reticulatus*	33119	5.3	5847	916
*Eremophila drummondii*	70212	4.8	10376	1943
*Eucryphia lucida*	35858	5.2	6865	1085
*Eupomatia laurina*	45657	4.6	12533	2879
*Flindersia xanthoxyla*	44496	5.5	6788	983
*Gastrolobium racemosum*	76884	4.7	9005	1806
*Gymnostoma australianum*	33918	5.2	8650	1376
*Hedycarya angustifolia*	36391	5.2	12054	2391
*Isopogon anethifolius*	40551	5.0	10113	1869
*Kingia australis*	56806	4.2	13076	3457
*Lepidosperma gladiatum*	24712	5.6	6500	950
*Leucochrysum albicans*	84045	4.8	8950	1675
*Nothofagus cunninghamii*	45938	4.8	8765	1642
*Pelargonium australe*	42215	5.2	7933	1215
*Phebalium stellatum*	47577	4.8	9002	1594
*Phyllanthus gunnii*	46606	4.5	9377	1855
*Pultenaea daphnoides*	47154	4.8	9905	1838
*Quintinia fawkneri*	50982	4.7	11490	2545
*Roepera similis*	51105	4.9	11946	2222
*Wahlenbergia ceracea*	51781	4.5	9282	1913

**Fig 5:**
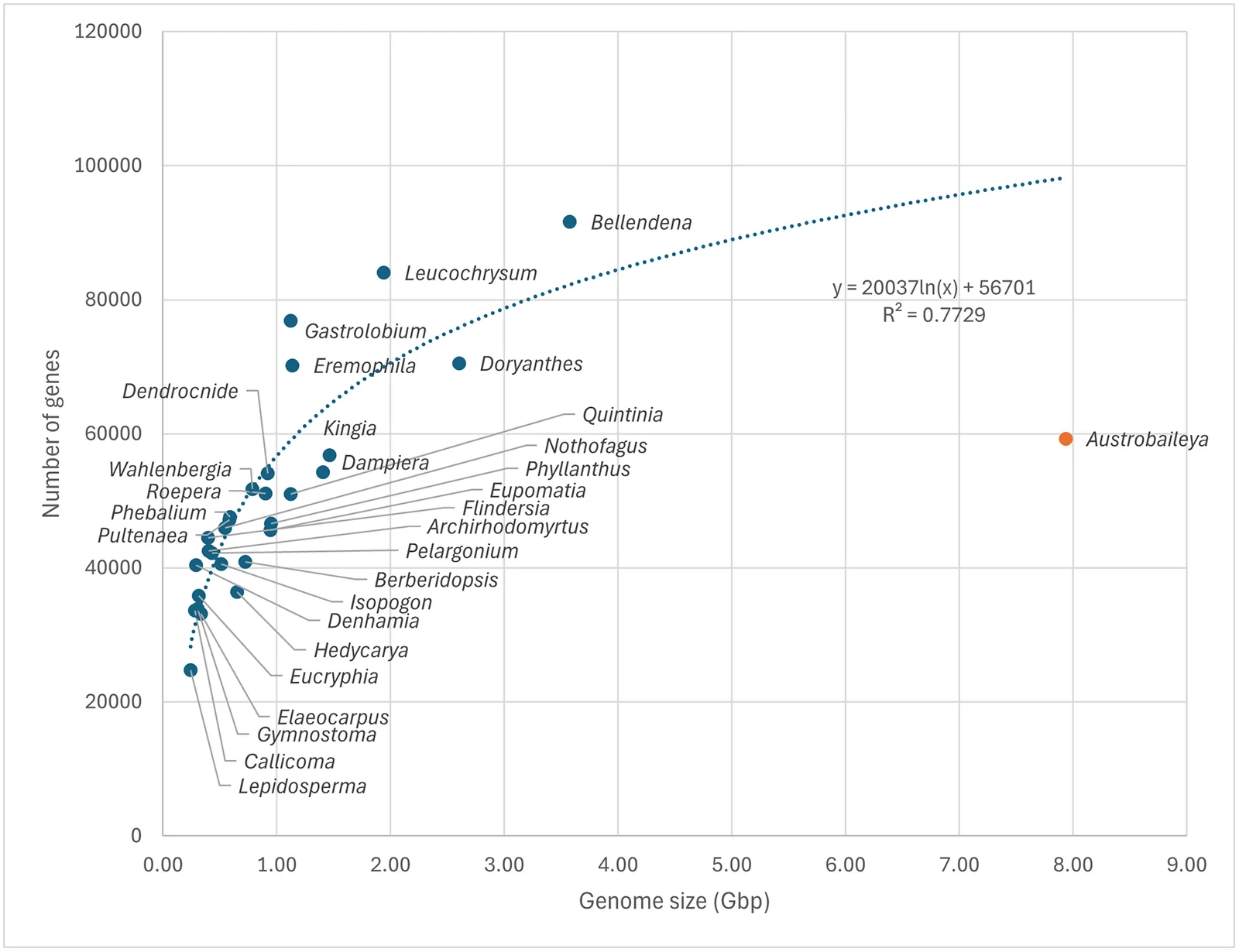
Number of genes annotated by GeMoMa Vs. observed haploid genome size. The trendline shown excluded *Austrobaileya scandens.*

The approach we have used – to map long reads to reference organelle genomes prior to separated nuclear and organelle genome assembly, appeared to have worked well, at least for plastid assembly (see Table 9). All plastid genomes were assembled to single contigs with the exception of *Roepera similis,* and *Flindersia xanthoxyla* (two contigs each). Mitochondrial genomes however, were more fragmented (1–12 contigs), and two assemblies, *Pelargonium australe* and *Gastrolobium racemosum* were exceptionally small and likely assembly failures. The sizes of plastid genomes (114–233 kbp for single contig) was within the expected range [67]. *Archirhodomyrtus beckleri* exhibited a single plastid contig of unusually large size, 233 kbp, which appears to be due to an expanded small single-copy (SSC) region. We did not attempt assembly of different plastid haplotypes as performed by [68]. The plastomes here therefore are only a representative structure. Similarly, mitomes are known to exist in many structural forms [69,70] and the assemblies here may only be a sample of the structural diversity present in each species. The organelle genome annotated gene content is summarised in a supplementary file (S3 File), which also provides gene counts per contig. Among plastomes, gene content was not consistent (98–174 genes). Genes that were absent in more than one species included: *infA* (absent in 12 species) which is known to be transferred to the nucleus in some angiosperms [71]; all *ndh* genes (absent in 2–5 species); and 11 ribosomal protein genes. A core of six *ndh* genes (*ndhD, ndhE, ndhF, ndhG, ndhH,* and *ndhI*) were absent in three plastomes (*Roepera similis, Eucryphia lucida,* and *Pultenaea daphnoides*). *Ndh* genes are known to have been lost in several groups of plants, including carnivores and heterotrophic plants, and they are now understood to be more dispensable than previously thought [72]. *Roepera similis* also lacks both *ycf1* and *ycf2* genes which are also often lost in heterotrophs [73]. Mitome gene content was more variable than plastomes (20–139 genes), reflecting the known diversity and complexity of plant mitomes [68].

**Table 9:** Organelle genome assembly details. Note – *Pelargonium australe* and *Gastrolobium racemosum* mitochondrion assemblies were much smaller than expected sizes and likely assembly failures.

Species	Plastid contigs	Plastid assembly size (bp) (all contigs)	Genes	Mitochondrial contigs	Mitochondrion assembly size (bp) (all contigs)	Genes
*Archirhodomyrtus beckleri*	1	232,601	174	4	791,470	87
*Austrobaileya scandens*	1	199,501	157	4	187,970	21
*Bellendena montana*	1	189,921	150	2	750,234	70
*Berberidopsis beckleri*	1	158,935	131	2	1,005,909	69
*Callicoma serratifolia*	1	177,987	161	4	564,896	63
*Dampiera purpurea*	1	155,834	126	3	298,696	50
*Dendrocnide excelsa*	1	181,980	145	6	406,345	109
*Denhamia bilocularis*	1	157,857	132	12	1,147,047	86
*Doryanthes excelsa*	1	159,193	133	3	1,016,924	86
*Elaeocarpus reticulatus*	1	145,780	122	1	368,431	37
*Eremophila drummondii*	1	151,677	128	3	767,416	95
*Eucryphia lucida*	1	114,022	98	2	626,021	80
*Eupomatia laurina*	1	159,718	130	4	297,146	20
*Flindersia xanthoxyla*	2	240,220	152	4	647,319	101
*Gastrolobium racemosum*	1	151,377	127	1	99,406	58
*Gymnostoma australianum*	1	157,744	130	1	513,370	47
*Hedycarya angustifolia*	1	182,055	139	6	707,125	48
*Isopogon anethifolius*	1	158,282	132	6	1,171,496	139
*Kingia australis*	1	195,884	158	2	741,578	88
*Lepidosperma gladiatum*	1	189,731	140	9	775,116	93
*Leucochrysum albicans*	1	154,047	129	4	409,322	75
*Nothofagus cunninghamii*	1	186,452	143	1	452,469	53
*Pelargonium australe*	1	153,769	124	1	49,781	1
*Phebalium stellatum*	1	180,664	143	6	579,814	83
*Phyllanthus gunnii*	1	180,174	143	4	257,208	31
*Pultenaea daphnoides*	1	141,812	118	1	425,848	48
*Quintinia fawkneri*	1	157,735	131	6	646,564	58
*Roepera similis*	2	181,130	120	2	670,751	64
*Wahlenbergia ceracea*	1	189,382	128	8	281,361	32

## Conclusion

These genomes represent a foundational resource for investigating the distinctive evolutionary radiations of the Australian flora, including adaptations to aridity, fire regimes, and nutrient-poor soils. By targeting phylogenetically significant lineages and species of conservation concern, this initiative addresses critical gaps in global plant genomic databases and establishes a framework for future population genomic and functional studies. The laboratory and bioinformatic methods applied here can be used for the future generation of high quality reference genomes in taxonomic groups still lacking published genomes, which were outside the scope of this study. All sequence data, annotations, and bioinformatics workflows are publicly available through NCBI and the project GitHub repository, supporting reproducible genomics research across the Australian botanical community and beyond.

## Supporting information

S1 File Genomescope kmer histogram plots. (XLSX)

S2 File nQuire results per contig. (XLSX)

S3 File Organelle annotations. (XLSX)

S1 Fig Kingia dot plots. (PNG)
