# Supplementary material for: Filling the gaps in Australian angiosperm genomes
Source: PLoS One. 2026 Nov 4;21(11):e0359340. doi: 10.1371/journal.pone.0359340 (PMC13734463; doi:10.1371/journal.pone.0359340)
Supplement: Supplementary file 1 [file pone.0359340.s006.docx]

**Filling the Gaps in Australian Angiosperm Genomes.**

**Response to Reviewers**

N.B. All line numbers refer to revised track changes version of the manuscript.

**Reviewer #1.**

*1) Please differentiate between genomes (DNA) and genome sequences (information). Unless a perfect representation is achieved, there might be fundamental differences (errors).*

We are not sure where in the manuscript these need to be differentiated? In general, the wording was used as following: 'Genome' - meaning the biological genetic content of the species; and 'genome sequences' being the DNA sequences that were derived from the genome. Since there is no way to know the genome without some imperfect method of reading it, we assume that the meaning of 'sequence' is implicit. If further elaboration is required, could the reviewer please provide the places in the manuscript where confusion arises?

*2) PRJEB88028 ... data is not accessible. Please check.*

Unfortunately, NCBI is currently experiencing long delays in review and publication of genome assemblies. The submitted assemblies are marked as 'processing', and have been for several months. There is no date embargo on the data and it will be available through NCBI as soon as they have processed it. In the meantime, I have also uploaded the assemblies to the Zenodo repository https://zenodo.org/records/20337510, and I have amended lines 280 and 306 accordingly. The sequence reads are available in the NCBI SRA if you search for the bioproject: https://www.ncbi.nlm.nih.gov/sra/?term=PRJNA1406336.

*3) https://zenodo.org/records/20337510 ... it seems that FASTA files with the genome sequences are missing here. This is a problem, because the GFF files provided here might not match the FASTA files provided by the NCBI.*

See 2) above.

*4) Hifi > HiFi. Careful proof reading by all authors is strongly recommended. There are some minor language issues.*

'Hifi' changed to 'HiFi' throughout.

*5) Line 70-73 could be more specific with numbers about the development. These studies might help: https://doi.org/10.1038/s41477-021-01031-8, https://doi.org/10.3389/fpls.2025.1603547, https://doi.org/10.1186/s12864-026-12623-z*

The authors feel that this statement stands as a general remark and does not require further elaboration because the subject of sequencing affordability is not a focus of the manuscript.

*6) Plastid and mitochondrial genomes -> plastome and chondrome. Since the genome is the total amount of DNA in a cell, the organelles could only harbour subgenomes.*

The authors regard 'Plastid and mitochondrial genomes' as valid and commonly used terms. Plastids and mitochondria are also often referred to as having their own genomes (e.g. see NCBI entries which are: "[Organism] mitochondrion, complete genome"), not just 'subgenomes' (which is a term less used). Also the word 'chondrome' is not commonly used and the authors prefer the use of 'mitochondrial genome' and / or 'mitome'. 'Plastome' and 'plastid genome' are also used interchangeably.

*7) "All sequence data, bioinformatic workflows, and specimen metadata are publicly available." ... this does not seem to be correct. Please see comment about accession above.*

See 2) above. As far as the authors are aware, all information is available.

*8) Dendrocnide excelsa -> The statement about Urticaceae is wrong, because there have been multiple genome sequencing projects in the family.*

The statement was changed in Table 1 from family to genus: "This species is a representative of the nettle family (Urticaceae) and the genome provides a reference for a previously unsampled genus."

*9) Table 1 should be checked again. There are many superficial statement that a reference sequences would be helpful without providing any specific applications/questions that would be addressed with it.*

We have edited Table 1 accordingly. The application / usefulness of reference genomes is addressed in the introduction final paragraph, lines 107 - 114. Therefore the individual superficial, general, statements referring to reference genomes in Table 1 have been removed as requested. However, references to specific unique applications of the reference genomes in Table 1 have been retained.

*10) "Young, healthy leaf material from the study species was harvested from actively growing plants then stored in a sealed bag at 4℃ until processing was possible" .... this seems to be problematic. How long was the material stored?*

Leaf was stored, at longest, overnight at 4C. This was not problematic for DNA preparation. Sufficient high molecular weight DNA was obtained for HiFi library preparation.

*11) "Dorado v7.3.11" ... please check this. The latest version should be v2.0 and that was released three weeks ago.*

Yes, this was a mistake, the version was 0.7.2, changed in line 198.

*12) genomescope ... check spelling throughout the manuscript*

We could not find a misspelling of 'genomescope' but we have changed it to the form used by the author, 'GenomeScope', on line 297 and 299.

*13) hifiasm references is missing in line 211.*

Added hifiasm reference.

*14) What is the difference between "genome ploidy" and "organismal ploidy"? Checking for consistency of terms might be helpful here.*

The terms are used interchangeably.

*15) "in the absence of trio samples (parents and offspring of controlled crosses) or Hi-C topological linking, the accurate production of haplomes is not possible" ... this is not correct. It can be possible to phase only based on long reads. However, it might not be possible in the species studied here or the coverage might be insufficient.*

We would argue that is not possible for accurate phasing using a single sample with no Hi-C in most plant species, although this could be a subjective matter. Plants have high repeat content in long stretches with low heterozygous content that can cause 'switch errors' and phasing to be incorrect even in high coverage assemblies. This is mentioned in Cheng et al., 2021 [52]. Further, the purpose of the assemblies' publication was not to provide phased haplotypes. If such are required then the vouchered specimens could be used for further experiments.

*16) Table 3: Some kind of per base accuracy could be included. Consider tools like Merqury (10.1186/s13059-020-02134-9). Also, the BUSCO results could be presented in this table using the summary string, because this would avoid the need for Table 5.*

Merqury analysis has been added to the study as requested. Per base quality was high for all sequence data (QV = 39 - 72). Completeness was also generally good (82 - 99%), *Dendrocnide* having the lowest value = 82% is now noted in the text, although we cannot currently comment on what cased the lower values in this species. We have now included Merqury results, line 244 and line 358; QV values and % completeness have been added to Table 5.

*17) "were likely to be telomere to telomere (T2T) complete chromosomes" ... they are likely to represent complete chromosomes*

Line 337 has been changed to, "Many contigs were likely to represent complete telomere to telomere (T2T) chromosomes".

*18) I would say that the assembly quality varies substantially. While there are some assemblies that look great based on the presented numbers, a BUSCO score of <95% would suggest a rather low completeness.*

Only one assembly BUSCO score, *Dendrocnide excelsa,* was below 95%. Line 354 has been changed to "The completeness of assemblies, as measured using BUSCO genes, was generally high, ranging from 99.88% for *Elaeocarpus reticulatus* to 93.12% for *Dendrocnide excelsa* (mean = 98.71%, stdev = 1.45, Table 5)."

*19) embryophyta_odb10.2019-11-20 is somewhat outdated as also indicated by the name. There is already v12 available.*

At the time of analysis the BUSCO database, 'embryophyta_odb10.2019-11-20', was the one available and included in the compleasm documentation. We do not feel that updating this and replicating the large analysis would appreciably alter results.

*20) Table 5 suggests that the haplo_purge did not work well. However, this could be an opportunity and the frequency of duplicated BUSCOs could support the ploidy analysis.*

In purging haplotigs, to some degree, a subjective threshold of alignment identity must be used to separate haplotypes but not remove contigs that are ancient duplications or contigs that are replicated blocks. The process is therefore not expected to be perfect, especially in complex plant genomes that may have undergone many ploidy events and / or be in the process of rediploidisation. To the best of our ability, we hope the manuscript has made this apparent in the discussion of BUSCO duplication and ploidy between lines 367 - 384 and Fig. 3.

*21) The huge variation in Table 8 could suggest that the filtering was not done properly for all species. This process should be described in more details in the method section to make it reproducible and consistent across all species. Based on available annotations, the average gene number in plants should be slightly below 30k (10.1186/s12864-018-5360-z). However, there is obviously a bias through the annotation process of all genome sequences considered in such studies. It would be important to present the gene number per haplophase, because polyploids would obviously have a multiple of that number.*

As above, it is not possible to distinguish genes that make up a haplophase in this analysis / assembly process, nor is it the aim of the study. As mentioned, where genomes contain stable duplications which may be rearranged among chromosomes, we feel it is not possible currently to assign a 'haplophase gene count' nor is it useful in the current paper. We present the annotations / gene counts as a comparative analysis among genomes that were assembled with as far as possible identical methods and annotation methods were identical. The wide variations can therefore be true features of the genomes, through duplication / ancient polyploid / rediploidisation. The genomes assembled and presented here represent more of a starting point resource for such studies - allowing e.g. comparative genomics as more genomes withing the taxonomic groups become available.

*22) The analysis of the mean gene length could be influenced by the parameters used during gene prediction. A larger max intron size could have resulted in a higher average gene size. If the mean gene length is a simple mean, it would be important to check if the underlying distribution is actually a normal distribution.*

The 'max intron size' was the default value for all GeMoMa annotations (15 kbp). We accept that deviation from normality would affect statistical tests, but none were performed among species in this study and the mean values are presented as indicative only of the potential true differences between species. A more in depth analysis of gene content is outside the scope of this paper.

*23) "Mitochondrial genomes however, were more fragmented (1 - 12 contigs)," ... could this be due to biology? There are hypotheses that the chondrome is actually present in multiple parts and not (always) a circular molecule.*

23) We acknowledge the variability in mitochondrial genome structure (line 467), as per the cited paper [68].

*24) "gene content was fairly consistent (126 -214 genes" ... these plastomes should be highly conserved with around 150 genes. This huge variation is surprising and should be investigated to rule out assembly/annotation artifacts.*

We agree that further investigation of the variation in plastomes would be interesting / useful, but with the data available for these assemblies it is not possible or in the scope of the paper. The plastomes are presented as a starting point for future research in these groups, when they could be confirmed / refuted by alternative and more in-depth methods tailored to each species. Please also note that in the cases of *Flindersia xanthoxyla* and *Roepera similis* the gene counts were across two contigs. We have expanded the assembly stats table in S3_Organelle_annotations.xlsx to include gene counts per contig for plastid and mitochondrial genomes and made a note in the text to this effect, Line 461. The plastid and mitochondrial assembly annotations have also been updated to a more recent analysis, which has altered the values in Table 9 and S3, but these have not altered the relevance of the reviewer's comments or the reply. The values have also been updated in the Results and Discussion paragraph, lines 448 - 473.

*25) While there is variation in "mitome gene content", it would be important to check if the numbers are consistent with the numbers reported for "closely" related species. This would help to rule out artifacts like the 26 genes observed in one species which seems too low for a plant.*

25) We agree, but answer as per 24).

*26) Fig.1: The assembly level classification into "contig", "scaffold", "chromosome" is misleading. For example, an assembly can be a perfect representation of a genome by having one contig per chromosome. Simultaneously, a highly fragmented and incomplete assembly could be scaffolded into sequences that are presented as pseudochromosomes. It would be more meaningful to use N50 or a similar metric for classification of assemblies.*

The assembly classification of "contig", "scaffold", and "chromosome" is that used by NCBI for completeness level of assemblies in the NCBI assembly / genome repository which is the source of the data, and this is stated in the legend. The Figure serves to illustrate the genomes available in these categories, not to comment on their relative completeness.

*27) Fig.6: The trend line is somewhat misleading here, because there are no reliable data points for half of the figure (>4Gbp).*

There is no Fig 6. If the reviewer meant Fig 2., then yes the data is sparse and all points are shown, so it is obvious the trend breaks down below 2 Gbp but there does appear to be a good correlation in general. As always, more data would make this more clear.

*28) Fig.2: This trend line shows a classical problem of enforcing a linear correlation although this might not describe the actual datapoints. There seems to be no correlation among most of the datapoints (estimated length <2Gbp) and only the three large "outliers" make it look like a linear correlation. I would recommend to check this again.*

As above, and the correlation is only presented as illustrative, we are not using it to model anything or make any predictions.

*29) S4: I do not see a clear pattern in here except for the match of each contig with itself.*

We agree, there are no clear patterns apart from contigs with themselves, which was the purpose of the figure, as described in lines 320 - 323. If haplotigs or homolgous blocks remained, they would likely be seen in the dot plots, assuming their contiguity and length was sufficient.

*30) S3: Maybe replace the GFF3 file name with the species name in the plastid genes table and the mitochondrion genes table.*

30) Done.

**Reviewer #2.**

*‘Under-represented orders 129 and families were targeted, with an emphasis on diploid genomes ≤ 2 Gb to enable high-quality assemblies’. Can authors explain why genomes <= 2Gb are easier to assemble at high-quality? I was expecting the reason to be budget-related. Also, larger genomes are included in the results.*

≤ 2 Gb allows for a reasonable sequencing depth ~20X with a single PacBio HiFi cell (approx. $10,000), so yes, it is a budgetary constraint.

*The GitHub repository has to be improved by providing a better README.md that specifies which analysis/scripts to run in which order.*

We hope that the methods section describes the programs that were run and the order. The GitHub readme has now been edited to include a description of the order of the scripts. The order itself is not critical, as most scripts are post-hoc analyses of the assembly, except the key feature that we employed, of separating organelle reads from nuclear reads before assembly, which is described in the methods, and now in the readme.

*‘Nuclear genomes were assembled with hifiasm v0.20.0-r639 [52] with the exception of 219 Callicoma serratifolia which was assembled using canu v2.3’. Why did C. serratifolia data return too few HERRO corrected reads?*

Unfortunately we do not know why *C. serratifolia*  reads did not produce enough HERRO corrected reads. We can assume that the raw ONT reads were of lower quality.

*Were assembly errors checked? It seems base-level accuracy is never measured (for example, with k-mer-based tools like Merqury or yak, or alignment-based tools like Inspector).*

See 16) for reviewer 1 above, Merqury % completeness and per base accuracy have now been added to the study and included in Table 5.

*‘Many contigs were likely to be telomere to telomere (T2T) complete chromosomes’. Why ‘likely’? Just because of the presence of terminal telomeres?*

Yes, the presence of terminal telomeres (and length) is a good indicator of a contig being a complete chromosome.

*Have the authors tried to compare all-vs-all the produced assemblies? An assembly graph could be prohibitive, given the likely sequence divergence, but alignment-based comparisons would be feasible and informative.*

We are not clear if the reviewer means all-vs-all among the different species - this would be extremely resource intensive and we cannot see any reason in the current scope for attempting this. If the reviewer means all-vs-all contigs within species, then we did perform this, as mentioned for *Kingia australis*, in which case it was performed to address a specific question of possible unpurged haplotigs. It was performed for other species to generally examine e.g. synteny / duplication between contigs in species that had apparently high duplication of genes after purging, but results are currently highly subjective. We feel this analysis would be best covered in subsequent studies of selected taxa, where synteny is a more pertinent question.

*Typo: the Bellendena gene count is 95,937 in the text but 91,604 in Table 8.*

Corrected in text.
