# Supplementary material for: Filling the gaps in Australian angiosperm genomes
Source: PLoS One. 2026 Nov 4;21(11):e0359340. doi: 10.1371/journal.pone.0359340 (PMC13734463; doi:10.1371/journal.pone.0359340)
Supplement: Peer review history

# Peer review: Decision Letter 0

**DOI:** 10.1371/journal.pone.0359340.r001

**Authors:** Muhammad Qasim Shahid

16 Jun 2026

Dear Dr. Allnutt,

Thank you for submitting your manuscript to PLOS ONE. After careful consideration, we feel that it has merit but does not fully meet PLOS ONE’s publication criteria as it currently stands. Therefore, we invite you to submit a revised version of the manuscript that addresses the points raised during the review process.

Please submit your revised manuscript by Aug 15 2026 11:59PM. If you will need more time than this to complete your revisions, please reply to this message or contact the journal office at plosone@plos.org. When you're ready to submit your revision, log on to https://www.editorialmanager.com/pone/ and select the 'Submissions Needing Revision' folder to locate your manuscript file.

- A letter that responds to each point raised by the academic editor and reviewer(s). You should upload this letter as a separate file labeled 'Response to Reviewers'.
- A marked-up copy of your manuscript that highlights changes made to the original version. You should upload this as a separate file labeled 'Revised Manuscript with Track Changes'.
- An unmarked version of your revised paper without tracked changes. You should upload this as a separate file labeled 'Manuscript'.

If you would like to make changes to your financial disclosure, please include your updated statement in your cover letter. Guidelines for resubmitting your figure files are available below the reviewer comments at the end of this letter.

If applicable, we recommend that you deposit your laboratory protocols in protocols.io to enhance the reproducibility of your results. Protocols.io assigns your protocol its own identifier (DOI) so that it can be cited independently in the future. For instructions see: https://journals.plos.org/plosone/s/submission-guidelines#loc-laboratory-protocols. Additionally, PLOS ONE offers an option for publishing peer-reviewed Lab Protocol articles, which describe protocols hosted on protocols.io. Read more information on sharing protocols at https://plos.org/protocols?utm_medium=editorial-email&utm_source=authorletters&utm_campaign=protocols.

As the corresponding author, your ORCID iD is verified in the submission system and will appear in the published article. PLOS supports the use of ORCID, and we encourage all coauthors to register for an ORCID iD and use it as well. Please encourage your coauthors to verify their ORCID iD within the submission system before final acceptance, as unverified ORCID iDs will not appear in the published article. *Only*  the individual author can complete the verification step; PLOS staff *cannot*  verify ORCID iDs on behalf of authors.

We look forward to receiving your revised manuscript.

Kind regards,

Muhammad Qasim Shahid

Academic Editor

PLOS One

Journal Requirements:

When submitting your revision, we need you to address these additional requirements.

1. Please ensure that your manuscript meets PLOS ONE’s style requirements, including those for file naming. The PLOS ONE style templates can be found at

https://journals.plos.org/plosone/s/file?id=wjVg/PLOSOne_formatting_sample_main_body.pdf and

https://journals.plos.org/plosone/s/file?id=ba62/PLOSOne_formatting_sample_title_authors_affiliations.pdf

2. Thank you for stating the following in the Acknowledgments Section of your manuscript:

“The Initiative is supported by funding from Bioplatforms Australia (enabled by NCRIS), the Ian Potter Foundation, Royal Botanic Gardens Foundation (Victoria), Royal Botanic Gardens Victoria, the Royal Botanic Gardens and Domain Trust, the Council of Heads of Australasian Herbaria, CSIRO, Centre for Australian National Biodiversity Research and the Department of Biodiversity, Conservation and Attractions, Western Australia. The authors acknowledge the provision of computing and data resources provided by the Australian BioCommons Leadership Share (ABLeS) program. The ABLeS program is co-funded by Bioplatforms Australia (enabled by NCRIS), the National Computational Infrastructure and Pawsey Supercomputing Research Centre.”

We note that you have provided funding information that is not currently declared in your Funding Statement. However, funding information should not appear in the Acknowledgments section or other areas of your manuscript. We will only publish funding information present in the Funding Statement section of the online submission form.

Please remove any funding-related text from the manuscript and let us know how you would like to update your Funding Statement. Currently, your Funding Statement reads as follows:

“The Initiative is supported by funding from Bioplatforms Australia (enabled by NCRIS), the Ian Potter Foundation, Royal Botanic Gardens Foundation (Victoria), Royal Botanic Gardens Victoria, the Royal Botanic Gardens and Domain Trust, the Council of Heads of Australasian Herbaria, CSIRO, Centre for Australian National Biodiversity Research and the Department of Biodiversity, Conservation and Attractions, Western Australia. The authors acknowledge the provision of computing and data resources provided by the Australian BioCommons Leadership Share (ABLeS) program. The ABLeS program is co-funded by Bioplatforms Australia (enabled by NCRIS), the National Computational Infrastructure and Pawsey Supercomputing Research Centre.”

Please include your amended statements within your cover letter; we will change the online submission form on your behalf.

3. Thank you for stating the following financial disclosure:

“The Initiative is supported by funding from Bioplatforms Australia (enabled by NCRIS), the Ian Potter Foundation, Royal Botanic Gardens Foundation (Victoria), Royal Botanic Gardens Victoria, the Royal Botanic Gardens and Domain Trust, the Council of Heads of Australasian Herbaria, CSIRO, Centre for Australian National Biodiversity Research and the Department of Biodiversity, Conservation and Attractions, Western Australia. The authors acknowledge the provision of computing and data resources provided by the Australian BioCommons Leadership Share (ABLeS) program. The ABLeS program is co-funded by Bioplatforms Australia (enabled by NCRIS), the National Computational Infrastructure and Pawsey Supercomputing Research Centre.”

Please state what role the funders took in the study.  If the funders had no role, please state: "The funders had no role in study design, data collection and analysis, decision to publish, or preparation of the manuscript."

If this statement is not correct you must amend it as needed.

Please include this amended Role of Funder statement in your cover letter; we will change the online submission form on your behalf.

4. Thank you for stating the following financial disclosure:

“The Initiative is supported by funding from Bioplatforms Australia (enabled by NCRIS), the Ian Potter Foundation, Royal Botanic Gardens Foundation (Victoria), Royal Botanic Gardens Victoria, the Royal Botanic Gardens and Domain Trust, the Council of Heads of Australasian Herbaria, CSIRO, Centre for Australian National Biodiversity Research and the Department of Biodiversity, Conservation and Attractions, Western Australia. The authors acknowledge the provision of computing and data resources provided by the Australian BioCommons Leadership Share (ABLeS) program. The ABLeS program is co-funded by Bioplatforms Australia (enabled by NCRIS), the National Computational Infrastructure and Pawsey Supercomputing Research Centre.”

We note that one or more of the authors is affiliated with the funding organization, indicating the funder may have had some role in the design, data collection, analysis or preparation of your manuscript for publication; in other words, the funder played an indirect role through the participation of the co-authors. If the funding organization did not play a role in the study design, data collection and analysis, decision to publish, or preparation of the manuscript and only provided financial support in the form of authors' salaries and/or research materials, please do the following:

1. Review your statements relating to the author contributions, and ensure you have specifically and accurately indicated the role(s) that these authors had in your study. These amendments should be made in the online form.

2. Confirm in your cover letter that you agree with the following statement, and we will change the online submission form on your behalf:

“The funder provided support in the form of salaries for authors [insert relevant initials], but did not have any additional role in the study design, data collection and analysis, decision to publish, or preparation of the manuscript. The specific roles of these authors are articulated in the ‘author contributions’ section.

5. If the reviewer comments include a recommendation to cite specific previously published works, please review and evaluate these publications to determine whether they are relevant and should be cited. There is no requirement to cite these works unless the editor has indicated otherwise.

Additional Editor Comments:

The manuscript’s results are interesting; however, reviewers have raised some concerns about the manuscript that must be clarified. So, this manuscript required a revision before being considered for publication in PLOS ONE. Please see the attached review reports for details.

[Note: HTML markup is below. Please do not edit.]

Reviewers' comments:

Reviewer’s Responses to Questions

**Comments to the Author**

1. Is the manuscript technically sound, and do the data support the conclusions?

Reviewer #1: Yes

Reviewer #2: Partly

**********

2. Has the statistical analysis been performed appropriately and rigorously?

Reviewer #1: N/A

Reviewer #2: N/A

**********

3. Have the authors made all data underlying the findings in their manuscript fully available?

The PLOS Data policy

Reviewer #1: No

Reviewer #2: Yes

**********

4. Is the manuscript presented in an intelligible fashion and written in standard English?

Reviewer #1: Yes

Reviewer #2: Yes

**********

Reviewer #1: Allnutt et al present the genome sequences of 29 Australian angiosperms. The manuscript presents background about the investigated species and statistics about the generated assemblies. The method section makes the workflows largely reproducible. Unfortunately, the actual genome sequences appear to be inaccessible at the moment. I believe this manuscript describes useful resource for the community. The quality of the assemblies based on long reads is overall high. However, there is no clear research question which might be problematic regarding the classification of this work as a research article. Please see some specific suggestions for further improvements below.

1) Please differentiate between genomes (DNA) and genome sequences (information). Unless a perfect representation is achieved, there might be fundamental differences (errors).

2) PRJEB88028 ... data is not accessible. Please check.

3) https://zenodo.org/records/20337510 ... it seems that FASTA files with the genome sequences are missing here. This is a problem, because the GFF files provided here might not match the FASTA files provided by the NCBI.

4) Hifi > HiFi. Careful proof reading by all authors is strongly recommended. There are some minor language issues.

5) Line 70-73 could be more specific with numbers about the development. These studies might help: https://doi.org/10.1038/s41477-021-01031-8, https://doi.org/10.3389/fpls.2025.1603547, https://doi.org/10.1186/s12864-026-12623-z

6) Plastid and mitochondrial genomes -> plastome and chondrome. Since the genome is the total amount of DNA in a cell, the organelles could only harbour subgenomes.

7) "All sequence data, bioinformatic workflows, and specimen metadata are publicly available." ... this does not seem to be correct. Please see comment about accession above.

8) Dendrocnide excelsa -> The statement about Urticaceae is wrong, because there have been multiple genome sequencing projects in the family.

9) Table 1 should be checked again. There are many superficial statement that a reference sequences would be helpful without providing any specific applications/questions that would be addressed with it.

10) "Young, healthy leaf material from the study species was harvested from actively growing plants then stored in a sealed bag at 4℃ until processing was possible" .... this seems to be problematic. How long was the material stored?

11) "Dorado v7.3.11" ... please check this. The latest version should be v2.0 and that was released three weeks ago.

12) genomescope ... check spelling throughout the manuscript

13) hifiasm references is missing in line 211.

14) What is the difference between "genome ploidy" and "organismal ploidy"? Checking for consistency of terms might be helpful here.

15) "in the absence of trio samples (parents and offspring of controlled crosses) or Hi-C topological linking, the accurate production of haplomes is not possible" ... this is not correct. It can be possible to phase only based on long reads. However, it might not be possible in the species studied here or the coverage might be insufficient.

16) Table 3: Some kind of per base accuracy could be included. Consider tools like Merqury (10.1186/s13059-020-02134-9). Also, the BUSCO results could be presented in this table using the summary string, because this would avoid the need for Table 5.

17) "were likely to be telomere to telomere (T2T) complete chromosomes" ... they are likely to represent complete chromosomes

18) I would say that the assembly quality varies substantially. While there are some assemblies that look great based on the presented numbers, a BUSCO score of <95% would suggest a rather low completeness.

19) embryophyta_odb10.2019-11-20 is somewhat outdated as also indicated by the name. There is already v12 available.

20) Table 5 suggests that the haplo_purge did not work well. However, this could be an opportunity and the frequency of duplicated BUSCOs could support the ploidy analysis.

21) The huge variation in Table 8 could suggest that the filtering was not done properly for all species. This process should be described in more details in the method section to make it reproducible and consistent across all species. Based on available annotations, the average gene number in plants should be slightly below 30k (10.1186/s12864-018-5360-z). However, there is obviously a bias through the annotation process of all genome sequences considered in such studies. It would be important to present the gene number per haplophase, because polyploids would obviously have a multiple of that number.

22) The analysis of the mean gene length could be influenced by the parameters used during gene prediction. A larger max intron size could have resulted in a higher average gene size. If the mean gene length is a simple mean, it would be important to check if the underlying distribution is actually a normal distribution.

23) "Mitochondrial genomes however, were more fragmented (1 - 12 contigs)," ... could this be due to biology? There are hypotheses that the chondrome is actually present in multiple parts and not (always) a circular molecule.

24) "gene content was fairly consistent (126 -214 genes" ... these plastomes should be highly conserved with around 150 genes. This huge variation is surprising and should be investigated to rule out assembly/annotation artifacts.

25) While there is variation in "mitome gene content", it would be important to check if the numbers are consistent with the numbers reported for "closely" related species. This would help to rule out artifacts like the 26 genes observed in one species which seems too low for a plant.

26) Fig.1: The assembly level classification into "contig", "scaffold", "chromosome" is misleading. For example, an assembly can be a perfect representation of a genome by having one contig per chromosome. Simultaneously, a highly fragmented and incomplete assembly could be scaffolded into sequences that are presented as pseudochromosomes. It would be more meaningful to use N50 or a similar metric for classification of assemblies.

27) Fig.6: The trend line is somewhat misleading here, because there are no reliable data points for half of the figure (>4Gbp).

28) Fig.2: This trend line shows a classical problem of enforcing a linear correlation although this might not describe the actual datapoints. There seems to be no correlation among most of the datapoints (estimated length <2Gbp) and only the three large "outliers" make it look like a linear correlation. I would recommend to check this again.

29) S4: I do not see a clear pattern in here except for the match of each contig with itself.

30) S3: Maybe replace the GFF3 file name with the species name in the plastid genes table and the mitochondrion genes table.

Reviewer #2: The authors present long-read assemblies for 29 Australian angiosperm species across 24 orders, with organelle assemblies, repeat and ploidy characterization, and annotation.

The paper is not so easy to read due to the number of tables and numbers. Data and results could be better described by working more on their visualization in the figures.

‘Under-represented orders 129 and families were targeted, with an emphasis on diploid genomes ≤ 2 Gb to enable high-quality assemblies’. Can authors explain why genomes <= 2Gb are easier to assemble at high-quality? I was expecting the reason to be budget-related. Also, larger genomes are included in the results.

The GitHub repository has to be improved by providing a better README.md that specifies which analysis/scripts to run in which order.

‘Nuclear genomes were assembled with hifiasm v0.20.0-r639 [52] with the exception of 219 Callicoma serratifolia which was assembled using canu v2.3’. Why did C. serratifolia data return too few HERRO corrected reads?

‘The bioinformatics pipeline detailed here was relatively straightforward, and assemblies did not require 'polishing' correction or downstream scaffolding steps due to the low error rate expected from Hifi technology and/or HERRO correction”. Were assembly errors checked? It seems base-level accuracy is never measured (for example, with k-mer-based tools like Merqury or yak, or alignment-based tools like Inspector).

‘Many contigs were likely to be telomere to telomere (T2T) complete chromosomes’. Why ‘likely’? Just because of the presence of terminal telomeres?

Have the authors tried to compare all-vs-all the produced assemblies? An assembly graph could be prohibitive, given the likely sequence divergence, but alignment-based comparisons would be feasible and informative.

Typo: the Bellendena gene count is 95,937 in the text but 91,604 in Table 8.

**********

what does this mean?). If published, this will include your full peer review and any attached files.

If you choose “no”, your identity will remain anonymous but your review may still be made public.

**Do you want your identity to be public for this peer review?**  For information about this choice, including consent withdrawal, please see our Privacy Policy

Reviewer #1: No

Reviewer #2: **Yes:**  Andrea Guarracino

**********

[NOTE: If reviewer comments were submitted as an attachment file, they will be attached to this email and accessible via the submission site. Please log into your account, locate the manuscript record, and check for the action link "View Attachments". If this link does not appear, there are no attachment files.]

To ensure your figures meet our technical requirements, please review our figure guidelines: https://journals.plos.org/plosone/s/figures

You may also use PLOS’s free figure tool, NAAS, to help you prepare publication quality figures: https://journals.plos.org/plosone/s/figures#loc-tools-for-figure-preparation.

NAAS will assess whether your figures meet our technical requirements by comparing each figure against our figure specifications.

# Author comment: Author response to Decision Letter 1

**DOI:** 10.1371/journal.pone.0359340.r002

24 Aug 2026

Filling the Gaps in Australian Angiosperm Genomes.

Response to Reviewers

N.B. All line numbers refer to revised track changes version of the manuscript.

Reviewer #1.

1) Please differentiate between genomes (DNA) and genome sequences (information). Unless a perfect representation is achieved, there might be fundamental differences (errors).

We are not sure where in the manuscript these need to be differentiated? In general, the wording was used as following: 'Genome' - meaning the biological genetic content of the species; and 'genome sequences' being the DNA sequences that were derived from the genome. Since there is no way to know the genome without some imperfect method of reading it, we assume that the meaning of 'sequence' is implicit. If further elaboration is required, could the reviewer please provide the places in the manuscript where confusion arises?

2) PRJEB88028 ... data is not accessible. Please check.

Unfortunately, NCBI is currently experiencing long delays in review and publication of genome assemblies. The submitted assemblies are marked as 'processing', and have been for several months. There is no date embargo on the data and it will be available through NCBI as soon as they have processed it. In the meantime, I have also uploaded the assemblies to the Zenodo repository https://zenodo.org/records/20337510, and I have amended lines 280 and 306 accordingly. The sequence reads are available in the NCBI SRA if you search for the bioproject: https://www.ncbi.nlm.nih.gov/sra/?term=PRJNA1406336.

3) https://zenodo.org/records/20337510 ... it seems that FASTA files with the genome sequences are missing here. This is a problem, because the GFF files provided here might not match the FASTA files provided by the NCBI.

See 2) above.

4) Hifi > HiFi. Careful proof reading by all authors is strongly recommended. There are some minor language issues.

'Hifi' changed to 'HiFi' throughout.

5) Line 70-73 could be more specific with numbers about the development. These studies might help: https://doi.org/10.1038/s41477-021-01031-8, https://doi.org/10.3389/fpls.2025.1603547, https://doi.org/10.1186/s12864-026-12623-z

The authors feel that this statement stands as a general remark and does not require further elaboration because the subject of sequencing affordability is not a focus of the manuscript.

6) Plastid and mitochondrial genomes -> plastome and chondrome. Since the genome is the total amount of DNA in a cell, the organelles could only harbour subgenomes.

The authors regard 'Plastid and mitochondrial genomes' as valid and commonly used terms. Plastids and mitochondria are also often referred to as having their own genomes (e.g. see NCBI entries which are: "[Organism] mitochondrion, complete genome"), not just 'subgenomes' (which is a term less used). Also the word 'chondrome' is not commonly used and the authors prefer the use of 'mitochondrial genome' and / or 'mitome'. 'Plastome' and 'plastid genome' are also used interchangeably.

7) "All sequence data, bioinformatic workflows, and specimen metadata are publicly available." ... this does not seem to be correct. Please see comment about accession above.

See 2) above. As far as the authors are aware, all information is available.

8) Dendrocnide excelsa -> The statement about Urticaceae is wrong, because there have been multiple genome sequencing projects in the family.

The statement was changed in Table 1 from family to genus: "This species is a representative of the nettle family (Urticaceae) and the genome provides a reference for a previously unsampled genus."

9) Table 1 should be checked again. There are many superficial statement that a reference sequences would be helpful without providing any specific applications/questions that would be addressed with it.

We have edited Table 1 accordingly. The application / usefulness of reference genomes is addressed in the introduction final paragraph, lines 107 - 114. Therefore the individual superficial, general, statements referring to reference genomes in Table 1 have been removed as requested. However, references to specific unique applications of the reference genomes in Table 1 have been retained.

10) "Young, healthy leaf material from the study species was harvested from actively growing plants then stored in a sealed bag at 4℃ until processing was possible" .... this seems to be problematic. How long was the material stored?

Leaf was stored, at longest, overnight at 4C. This was not problematic for DNA preparation. Sufficient high molecular weight DNA was obtained for HiFi library preparation.

11) "Dorado v7.3.11" ... please check this. The latest version should be v2.0 and that was released three weeks ago.

Yes, this was a mistake, the version was 0.7.2, changed in line 198.

12) genomescope ... check spelling throughout the manuscript

We could not find a misspelling of 'genomescope' but we have changed it to the form used by the author, 'GenomeScope', on line 297 and 299.

13) hifiasm references is missing in line 211.

Added hifiasm reference.

14) What is the difference between "genome ploidy" and "organismal ploidy"? Checking for consistency of terms might be helpful here.

The terms are used interchangeably.

15) "in the absence of trio samples (parents and offspring of controlled crosses) or Hi-C topological linking, the accurate production of haplomes is not possible" ... this is not correct. It can be possible to phase only based on long reads. However, it might not be possible in the species studied here or the coverage might be insufficient.

We would argue that is not possible for accurate phasing using a single sample with no Hi-C in most plant species, although this could be a subjective matter. Plants have high repeat content in long stretches with low heterozygous content that can cause 'switch errors' and phasing to be incorrect even in high coverage assemblies. This is mentioned in Cheng et al., 2021 [52]. Further, the purpose of the assemblies' publication was not to provide phased haplotypes. If such are required then the vouchered specimens could be used for further experiments.

16) Table 3: Some kind of per base accuracy could be included. Consider tools like Merqury (10.1186/s13059-020-02134-9). Also, the BUSCO results could be presented in this table using the summary string, because this would avoid the need for Table 5.

Merqury analysis has been added to the study as requested. Per base quality was high for all sequence data (QV = 39 - 72). Completeness was also generally good (82 - 99%), Dendrocnide having the lowest value = 82% is now noted in the text, although we cannot currently comment on what cased the lower values in this species. We have now included Merqury results, line 244 and line 358; QV values and % completeness have been added to Table 5.

17) "were likely to be telomere to telomere (T2T) complete chromosomes" ... they are likely to represent complete chromosomes

Line 337 has been changed to, "Many contigs were likely to represent complete telomere to telomere (T2T) chromosomes".

18) I would say that the assembly quality varies substantially. While there are some assemblies that look great based on the presented numbers, a BUSCO score of <95% would suggest a rather low completeness.

Only one assembly BUSCO score, Dendrocnide excelsa, was below 95%. Line 354 has been changed to "The completeness of assemblies, as measured using BUSCO genes, was generally high, ranging from 99.88% for Elaeocarpus reticulatus to 93.12% for Dendrocnide excelsa (mean = 98.71%, stdev = 1.45, Table 5)."

19) embryophyta_odb10.2019-11-20 is somewhat outdated as also indicated by the name. There is already v12 available.

At the time of analysis the BUSCO database, 'embryophyta_odb10.2019-11-20', was the one available and included in the compleasm documentation. We do not feel that updating this and replicating the large analysis would appreciably alter results.

20) Table 5 suggests that the haplo_purge did not work well. However, this could be an opportunity and the frequency of duplicated BUSCOs could support the ploidy analysis.

In purging haplotigs, to some degree, a subjective threshold of alignment identity must be used to separate haplotypes but not remove contigs that are ancient duplications or contigs that are replicated blocks. The process is therefore not expected to be perfect, especially in complex plant genomes that may have undergone many ploidy events and / or be in the process of rediploidisation. To the best of our ability, we hope the manuscript has made this apparent in the discussion of BUSCO duplication and ploidy between lines 367 - 384 and Fig. 3.

21) The huge variation in Table 8 could suggest that the filtering was not done properly for all species. This process should be described in more details in the method section to make it reproducible and consistent across all species. Based on available annotations, the average gene number in plants should be slightly below 30k (10.1186/s12864-018-5360-z). However, there is obviously a bias through the annotation process of all genome sequences considered in such studies. It would be important to present the gene number per haplophase, because polyploids would obviously have a multiple of that number.

As above, it is not possible to distinguish genes that make up a haplophase in this analysis / assembly process, nor is it the aim of the study. As mentioned, where genomes contain stable duplications which may be rearranged among chromosomes, we feel it is not possible currently to assign a 'haplophase gene count' nor is it useful in the current paper. We present the annotations / gene counts as a comparative analysis among genomes that were assembled with as far as possible identical methods and annotation methods were identical. The wide variations can therefore be true features of the genomes, through duplication / ancient polyploid / rediploidisation. The genomes assembled and presented here represent more of a starting point resource for such studies - allowing e.g. comparative genomics as more genomes withing the taxonomic groups become available.

22) The analysis of the mean gene length could be influenced by the parameters used during gene prediction. A larger max intron size could have resulted in a higher average gene size. If the mean gene length is a simple mean, it would be important to check if the underlying distribution is actually a normal distribution.

The 'max intron size' was the default value for all GeMoMa annotations (15 kbp). We accept that deviation from normality would affect statistical tests, but none were performed among species in this study and the mean values are presented as indicative only of the potential true differences between species. A more in depth analysis of gene content is outside the scope of this paper.

23) "Mitochondrial genomes however, were more fragmented (1 - 12 contigs)," ... could this be due to biology? There are hypotheses that the chondrome is actually present in multiple parts and not (always) a circular molecule.

23) We acknowledge the variability in mitochondrial genome structure (line 467), as per the cited paper [68].

24) "gene content was fairly consistent (126 -214 genes" ... these plastomes should be highly conserved with around 150 genes. This huge variation is surprising and should be investigated to rule out assembly/annotation artifacts.

We agree that further investigation of the variation in plastomes would be interesting / useful, but with the data available for these assemblies it is not possible or in the scope of the paper. The plastomes are presented as a starting point for future research in these groups, when they could be confirmed / refuted by alternative and more in-depth methods tailored to each species. Please also note that in the cases of Flindersia xanthoxyla and Roepera similis the gene counts were across two contigs. We have expanded the assembly stats table in S3_Organelle_annotations.xlsx to include gene counts per contig for plastid and mitochondrial genomes and made a note in the text to this effect, Line 461. The plastid and mitochondrial assembly annotations have also been updated to a more recent analysis, which has altered the values in Table 9 and S3, but these have not altered the relevance of the reviewer’s comments or the reply. The values have also been updated in the Results and Discussion paragraph, lines 448 - 473.

25) While there is variation in "mitome gene content", it would be important to check if the numbers are consistent with the numbers reported for "closely" related species. This would help to rule out artifacts like the 26 genes observed in one species which seems too low for a plant.

25) We agree, but answer as per 24).

26) Fig.1: The assembly level classification into "contig", "scaffold", "chromosome" is misleading. For example, an assembly can be a perfect representation of a genome by having one contig per chromosome. Simultaneously, a highly fragmented and incomplete assembly could be scaffolded into sequences that are presented as pseudochromosomes. It would be more meaningful to use N50 or a similar metric for classification of assemblies.

The assembly classification of "contig", "scaffold", and "chromosome" is that used by NCBI for completeness level of assemblies in the NCBI assembly / genome repository which is the source of the data, and this is stated in the legend. The Figure serves to illustrate the genomes available in these categories, not to comment on their relative completeness.

27) Fig.6: The trend line is somewhat misleading here, because there are no reliable data points for half of the figure (>4Gbp).

There is no Fig 6. If the reviewer meant Fig 2., then yes the data is sparse and all points are shown, so it is obvious the trend breaks down below 2 Gbp but there does appear to be a good correlation in general. As always, more data would make this more clear.

28) Fig.2: This trend line shows a classical problem of enforcing a linear correlation although this might not describe the actual datapoints. There seems to be no correlation among most of the datapoints (estimated length <2Gbp) and only the three large "outliers" make it look like a linear correlation. I would recommend to check this again.

As above, and the correlation is only presented as illustrative, we are not using it to model anything or make any predictions.

29) S4: I do not see a clear pattern in here except for the match of each contig with itself.

We agree, there are no clear patterns apart from contigs with themselves, which was the purpose of the figure, as described in lines 320 - 323. If haplotigs or homolgous blocks remained, they would likely be seen in the dot plots, assuming their contiguity and length was sufficient.

30) S3: Maybe replace the GFF3 file name with the species name in the plastid genes table and the mitochondrion genes table.

30) Done.

Reviewer #2.

‘Under-represented orders 129 and families were targeted, with an emphasis on diploid genomes ≤ 2 Gb to enable high-quality assemblies’. Can authors explain why genomes <= 2Gb are easier to assemble at high-quality? I was expecting the reason to be budget-related. Also, larger genomes are included in the results.

≤ 2 Gb allows for a reasonable sequencing depth ~20X with a single PacBio HiFi cell (approx. $10,000), so yes, it is a budgetary constraint.

The GitHub repository has to be improved by providing a better README.md that specifies which analysis/scripts to run in which order.

We hope that the methods section describes the programs that were run and the order. The GitHub readme has now been edited to include a description of the order of the scripts. The order itself is not critical, as most scripts are post-hoc analyses of the assembly, except the key feature that we employed, of separating organelle reads from nuclear reads before assembly, which is described in the methods, and now in the readme.

‘Nuclear genomes were assembled with hifiasm v0.20.0-r639 [52] with the exception of 219 Callicoma serratifolia which was assembled using canu v2.3’. Why did C. serratifolia data return too few HERRO corrected reads?

Unfortunately we do not know why C. serratifolia reads did not produce enough HERRO corrected reads. We can assume that the raw ONT reads were of lower quality.

Were assembly errors checked? It seems base-level accuracy is never measured (for example, with k-mer-based tools like Merqury or yak, or alignment-based tools like Inspector).

See 16) for reviewer 1 above, Merqury % completeness and per base accuracy have now been added to the study and included in Table 5.

‘Many contigs were likely to be telomere to telomere (T2T) complete chromosomes’. Why ‘likely’? Just because of the presence of terminal telomeres?

Yes, the presence of terminal telomeres (and length) is a good indicator of a contig being a complete chromosome.

Have the authors tried to compare all-vs-all the produced assemblies? An assembly graph could be prohibitive, given the likely sequence divergence, but alignment-based comparisons would be feasible and informative.

We are not clear if the reviewer means all-vs-all among the different species - this would be extremely resource intensive and we cannot see any reason in the current scope for attempting this. If the reviewer means all-vs-all contigs within species, then we did perform this, as mentioned for Kingia australis, in which case it was performed to address a specific question of possible unpurged haplotigs. It was performed for other species to generally examine e.g. synteny / duplication between contigs in species that had apparently high duplication of genes after purging, but results are currently highly subjective. We feel this analysis would be best covered in subsequent studies of selected taxa, where synteny is a more pertinent question.

Typo: the Bellendena gene count is 95,937 in the text but 91,604 in Table 8.

Corrected in text.

Attachment Submitted filename: Response_to_Reviewers.docx

# Peer review: Decision Letter 1

**DOI:** 10.1371/journal.pone.0359340.r003

**Authors:** Muhammad Qasim Shahid

13 Sep 2026

<p>Filling the Gaps in Australian Angiosperm Genomes.

PONE-D-26-25929R1

Dear Dr. Allnutt,

We’re pleased to inform you that your manuscript has been judged scientifically suitable for publication and will be formally accepted for publication once it meets all outstanding technical requirements.

Within one week, you’ll receive an e-mail detailing the required amendments. When these have been addressed, you’ll receive a formal acceptance letter and your manuscript will be scheduled for publication.

An invoice will be generated when your article is formally accepted. Please note, if your institution has a publishing partnership with PLOS and your article meets the relevant criteria, all or part of your publication costs will be covered. Please make sure your user information is up-to-date by logging into Editorial Manager at Editorial Manager® and clicking the ‘Update My Information' link at the top of the page. For questions related to billing, please contact billing support.

If your institution or institutions have a press office, please notify them about your upcoming paper to help maximize its impact. If they’ll be preparing press materials, please inform our press team as soon as possible -- no later than 48 hours after receiving the formal acceptance. Your manuscript will remain under strict press embargo until 2 pm Eastern Time on the date of publication. For more information, please contact onepress@plos.org.

Kind regards,

Muhammad Qasim Shahid

Academic Editor

PLOS One

Additional Editor Comments (optional):

Please cross-check species names during proofreading

Reviewers' comments:

Reviewer’s Responses to Questions

**Comments to the Author**

Reviewer #2: All comments have been addressed

Reviewer #3: All comments have been addressed

**********

2. Is the manuscript technically sound, and do the data support the conclusions?

Reviewer #2: Yes

Reviewer #3: Yes

**********

3. Has the statistical analysis been performed appropriately and rigorously?

Reviewer #2: N/A

Reviewer #3: (No Response)

**********

4. Have the authors made all data underlying the findings in their manuscript fully available?

The PLOS Data policy

Reviewer #2: Yes

Reviewer #3: (No Response)

**********

5. Is the manuscript presented in an intelligible fashion and written in standard English?

Reviewer #2: Yes

Reviewer #3: Yes

**********

Reviewer #2: Authors addressed all my comments, I don't have anything else to report to them. .

Reviewer #3: Authors have addressed the comments. Overall, ms well written and could be accepted for publication. However, authros can cross-check species names during proofreading, such as • Phyllanthus gunnii / Lysiandra gunnii ; Gastrolobium racemosum versus the apparent “Gastrolobium australianum” mention in the organelle section.

**********

what does this mean?). If published, this will include your full peer review and any attached files.

If you choose “no”, your identity will remain anonymous but your review may still be made public.

**Do you want your identity to be public for this peer review?**  For information about this choice, including consent withdrawal, please see our Privacy Policy

Reviewer #2: **Yes:**  Andrea Guarracino

Reviewer #3: No

**********

# Editor report: Acceptance letter

**DOI:** 10.1371/journal.pone.0359340.r004

**Authors:** Muhammad Qasim Shahid

PONE-D-26-25929R1

PLOS One

Dear Dr. Allnutt,

I'm pleased to inform you that your manuscript has been deemed suitable for publication in PLOS One. Congratulations! Your manuscript is now being handed over to our production team.

At this stage, our production department will prepare your paper for publication. This includes ensuring the following:

* All references, tables, and figures are properly cited

* All relevant supporting information is included in the manuscript submission,

* There are no issues that prevent the paper from being properly typeset

You will receive further instructions from the production team, including instructions on how to review your proof when it is ready. Please keep in mind that we are working through a large volume of accepted articles, so please give us a few days to review your paper and let you know the next and final steps.

Lastly, if your institution or institutions have a press office, please let them know about your upcoming paper now to help maximize its impact. If they'll be preparing press materials, please inform our press team within the next 48 hours. Your manuscript will remain under strict press embargo until 2 pm Eastern Time on the date of publication. For more information, please contact onepress@plos.org.

You will receive an invoice from PLOS for your publication fee after your manuscript has reached the completed accept phase. If you receive an email requesting payment before acceptance or for any other service, this may be a phishing scheme. Learn how to identify phishing emails and protect your accounts at https://explore.plos.org/phishing.

If we can help with anything else, please email us at customercare@plos.org.

Thank you for submitting your work to PLOS One and supporting open access.

Kind regards,

PLOS One Editorial Office Staff

on behalf of

Dr. Muhammad Qasim Shahid

Academic Editor

PLOS One
